# Increased blood draws for ultrasensitive ctDNA and CTCs detection in early breast cancer patients

**DOI:** 10.1038/s41523-024-00642-6

**Published:** 2024-05-15

**Authors:** Alfonso Alba-Bernal, Ana Godoy-Ortiz, María Emilia Domínguez-Recio, Esperanza López-López, María Elena Quirós-Ortega, Victoria Sánchez-Martín, María Dunia Roldán-Díaz, Begoña Jiménez-Rodríguez, Jesús Peralta-Linero, Estefanía Bellagarza-García, Laura Troyano-Ramos, Guadalupe Garrido-Ruiz, M. Isabel Hierro-Martín, Luis Vicioso, Álvaro González-Ortiz, Noelia Linares-Valencia, Jesús Velasco-Suelto, Guillermo Carbajosa, Alicia Garrido-Aranda, Rocío Lavado-Valenzuela, Martina Álvarez, Javier Pascual, Iñaki Comino-Méndez, Emilio Alba

**Affiliations:** 1 Unidad de Gestion Clinica Intercentros de Oncologia Medica, Hospitales Universitarios Regional y Virgen de la Victoria, 29010 Malaga, Spain; 2 The Biomedical Research Institute of Málaga (IBIMA-CIMES-UMA), 29010 Malaga, Spain; 3 Andalusia-Roche Network in Precision Medical Oncology, 41092 Sevilla, Spain; 4 Centro de Investigacion Biomedica en Red de Cancer (CIBERONC - CB16/12/00481), 28029 Madrid, Spain; 5 Radiology Department, Hospital Clinico Universitario Virgen de la Victoria de Malaga, 29010 Malaga, Spain; 6 Unidad de Gestion Clinica Provincial de Anatomia Patologica de Malaga, Hospital Clinico Universitario Virgen de la Victoria de Malaga, 29010 Malaga, Spain; 7 University of Málaga, Faculty of Medicine, 29010 Malaga, Spain; 8 Laboratorio de biologia molecular del cancer (LBMC), Centro de investigaciones medico-sanitarias (CIMES-UMA), 29010 Malaga, Spain

**Keywords:** Breast cancer, Predictive markers, Prognostic markers

## Abstract

Early breast cancer patients often experience relapse due to residual disease after treatment. Liquid biopsy is a methodology capable of detecting tumor components in blood, but low concentrations at early stages pose challenges. To detect them, next-generation sequencing has promise but entails complex processes. Exploring larger blood volumes could overcome detection limitations. Herein, a total of 282 high-volume plasma and blood-cell samples were collected for dual ctDNA/CTCs detection using a single droplet-digital PCR assay per patient. ctDNA and/or CTCs were detected in 100% of pre-treatment samples. On the other hand, post-treatment positive samples exhibited a minimum variant allele frequency of 0.003% for ctDNA and minimum cell number of 0.069 CTCs/mL of blood, surpassing previous investigations. Accurate prediction of residual disease before surgery was achieved in patients without a complete pathological response. A model utilizing ctDNA dynamics achieved an area under the ROC curve of 0.92 for predicting response. We detected disease recurrence in blood in the three patients who experienced a relapse, anticipating clinical relapse by 34.61, 9.10, and 7.59 months. This methodology provides an easily implemented alternative for ultrasensitive residual disease detection in early breast cancer patients.

## Introduction

Breast cancer (BC) is a prevalent cancer among women in Western society. While early detection through screening guidelines has improved mortality rates^1^, approximately 20% of patients diagnosed at early stages experience relapse with incurable metastatic disease. This is mainly due to residual disease (RD) remaining after standard primary treatments. (Neo)adjuvant chemotherapy (NAC) is administered to BC patients with localized or locally advanced tumors before surgical removal. Pathological complete response (PCR) in the surgical specimen is used to assess treatment response^2,3^, but its limitations^4,5^ necessitate the discovery of new methodologies to predict RD and stratify patients for relapse risk. Accurate assessment of therapy response prior to surgery and detection of RD could lead to less radical treatment interventions^6–8^.

Invasive tumor biopsies are traditionally required for definitive BC diagnosis, posing risks and discomfort to patients^9^. Liquid biopsy has emerged as a non-invasive alternative to determine the presence of disease through the detection of tumor components in biofluids such as blood. Circulating tumor DNA (ctDNA) and circulating tumor cells (CTCs) are the most studied circulating tumor components in BC blood samples. However, their low concentrations in patients with localized tumors make detection challenging^10–12^.

Advanced next-generation sequencing (NGS) technologies have been developed to detect ctDNA with high sensitivity^13–16^, surpassing droplet-digital PCR (ddPCR) approaches^17–21^. Yet, their clinical application is complicated by the need for patient-specific panels and intensive sequencing processes. Although these technologies are accessible through service requests from private companies, their current costs pose a barrier to clinical implementation across the different healthcare systems. Moreover, the rarity of these components hampers their detection and characterization using conventional blood volumes. Exploring increased blood volumes as a potential solution to detection challenges remains unstudied^12,22–24^.

In this proof-of-concept study, we have developed an easily implementable and highly sensitive methodology for detecting circulating tumor components in early-stage BC patients. This innovative approach involves utilizing larger blood volumes and employing highly partitioned ddPCR assays to detect both ctDNA and CTCs, targeting a specific truncal somatic mutation for each patient. Our investigation primarily focused on assessing blood RD after NAC and prior to surgery and minimal residual disease (MRD) after surgery to accurately predict treatment response and molecular relapse before clinically evident.

## Results

### Methodology optimization for plasma DNA isolation and CTCs detection

In this study, a novel DNA extraction procedure^25^ was applied to extract DNA from 20 mL of plasma, achieving higher purity and lower germline contamination (Supplementary Fig. 1). Additionally, a mimicry experiment was performed to optimize the extraction procedure for CTCs and quantify them per mL of blood. The experiment involved spiking PBMCs with serial dilutions of MCF7 cells and using negative selection to enrich for them. A ddPCR assay targeting a specific gene mutation was optimized (Supplementary Fig. 2A) where the heterozygosity of the *PIK3CA* p.E545K mutation was re-validated (69.4 copies/μl of the mutant and 50 copies/μl of the wild-type alleles). Highly accurate linear regression (R^2^ = 0.9952) allowed the inference of CTCs count in patients’ samples (Supplementary Table 1, Supplementary Fig. 2B).

**Fig. 1:**
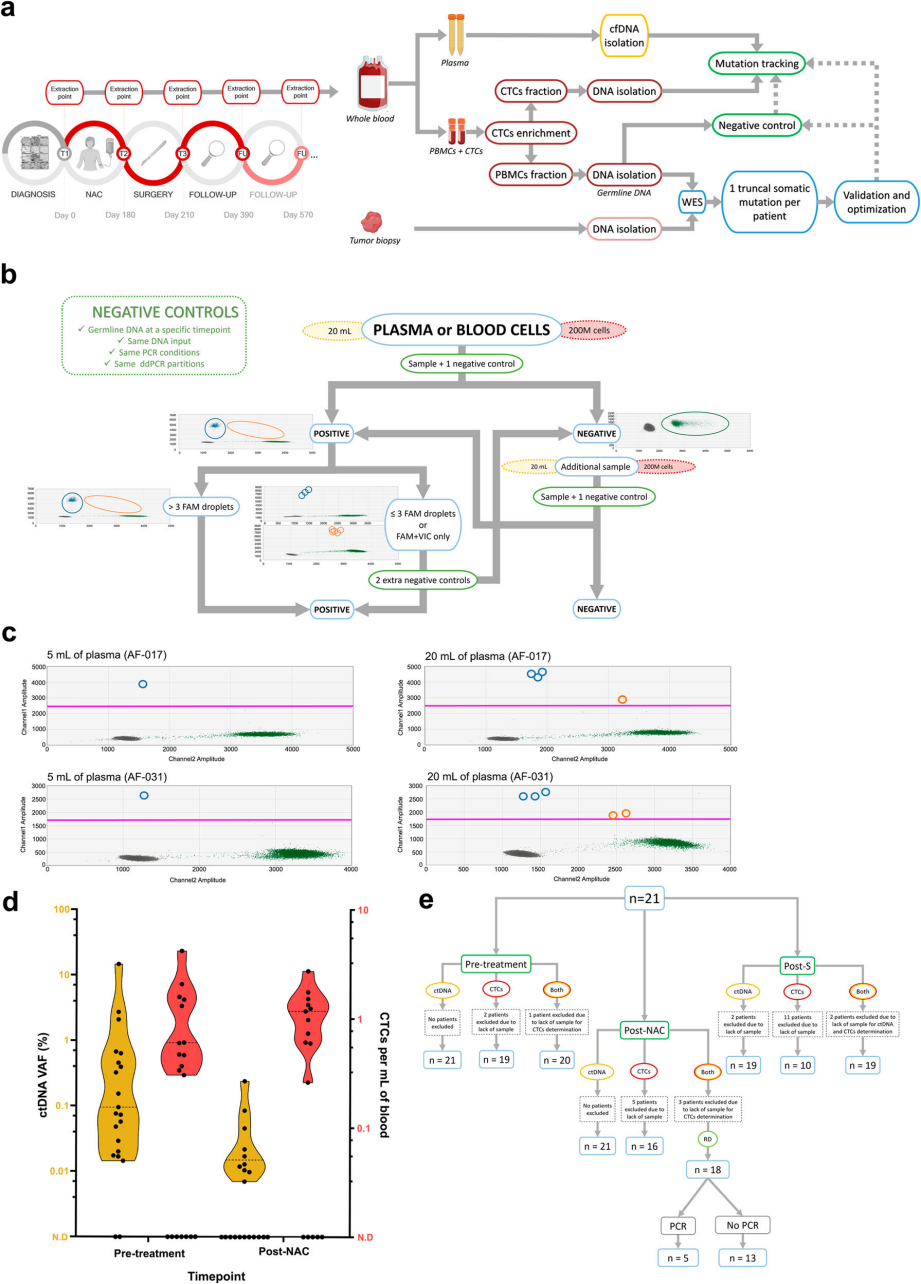
Workflow, assay optimization, and detection of ctDNA/CTCs in pre- and post-NAC blood samples. **a** Schema illustrating the study workflow, starting with blood sample collection at breast cancer diagnosis (T1) and subsequent samples after neoadjuvant chemotherapy (NAC) (T2), after surgery (T3), and during follow-up (T4). The diagram also depicts the steps involved in sample processing. **b** Diagram representing the steps for detecting the presence of ctDNA or CTCs in a plasma or blood cell sample, with specifications provided for the negative controls. **c** Examples demonstrating improved ctDNA detection in pre-treatment plasma samples using high plasma volumes compared to conventional methods. Mutant droplets are represented by blue dots (FAM-labeled), wild-type droplets by green dots (VIC-labeled), and droplets containing both wildtype and mutant molecules by orange dots. The pink line indicates the threshold for considering FAM-positive droplets. **d** Violin plots displaying individual values and median variant allele frequencies (VAFs) for ctDNA (orange) and CTCs (red) in pre- and post-NAC blood samples (medians are illustrated as dashed lines and the upper and lower limits of the plots representing the maximum and minimum values, respectively). **e** Flow chart depicting the number of eligible patients selected for each analysis. NAC Neoadjuvant chemotherapy, PBMCs Peripheral blood mononuclear cells, cfDNA circulating free DNA, ctDNA circulating tumor DNA, CTCs circulating tumor cells, WES Whole exome sequencing, RD Residual disease.

This methodology was applied to detect ctDNA and CTCs in samples from 21 early BC patients treated with NAC (Table 1). A total of 182 plasma samples and 100 PBMCs samples were extracted before, after NAC, 1 month after surgery, and every 6 months in those with the highest risk of relapse patients (Fig. 1a, Supplementary Table 2). The goal was to assay first a minimum of 20 mL of plasma for ctDNA detection and a minimum of 2 vials containing 100 million PBMCs each for CTCs identification. A total of 337 negative controls utilizing the corresponding patients’ germline DNA were employed to eliminate false negatives and ensure ultra-sensitive detection (Fig. 1b).

**Table 1:** Clinicopathological characteristics of the patients at the pre-treatment setting

Clinical characteristics		*n* (%)
Diagnostic age (years)		
<50		9 (43%)
>50		12 (57%)
Histological subtype		
IDC		20 (95%)
ILC		1 (5%)
Tumor subtype		
Luminal		13 (62%)
Triple negative		7 (33%)
HER2-enriched		1 (5%)
Tumor grade		
2		5 (24%)
3		16 (76%)
TNM		
IIA	cT2N0	8 (38%)
IIB	cT2N1	6 (29%)
	cT3N0	3 (14%)
IIIA	ct2N2	2 (9%)
	cT3N1	1 (5%)
IIIB	cT4bN1	1 (5%)
Axillar lymph node		
N0		12 (57%)
N1		6 (29%)
N2		2 (9%)
N3		1 (5%)
Estrogenic receptor		
Positive		13 (62%)
Negative		8 (38%)
Progesterone receptor		
Positive		8 (38%)
Negative		13 (62%)
HER2 status		
Positive		11 (52%)
Negative		10 (48%)
BIRADS category		
4/B/C		6 (29%)
5 C		15 (71%)
PCR		
Yes		7 (33%)
No		14 (67%)
Clinical relapse		
Yes		3 (14%)
No		18 (86%)

*IDC* invasive ductal carcinoma, *ILC* invasive lobular carcinoma, *N0* no lymph nodes affected, *N1-N3* from 1 to 10 or more lymph nodes affected, *TNM* tumor, node, metastasis, *PCR* pathological complete response.

### Pre-treatment detection of ctDNA and CTCs in early BC patients

Pre-treatment samples were analyzed for ctDNA and CTCs detection, using WES and RNAseq to identify patient-specific mutations (Fig. 1a). A median of 15 mutations (range 5–301) per patient was detected in the WES analysis of 19 tumor biopsies and corresponding germline DNA (Supplementary Table 3). One truncal somatic mutation per patient was selected as a biomarker for ctDNA and CTCs detection, re-validated as somatic, and optimized for ddPCR (Supplementary Fig. 3, Supplementary Table 4). The copy number variation (CNV) status for a given selected mutation can influence the extrapolation for CTCs determination using the MFC7 model described herein, specifically if the mutation allele experiences copy gains. To address this, we employed the CNV kit software to investigate the CNV affecting the mutations with higher VAFs in the WES and ddPCR experiments, the samples AF-059 and AF-085 (with VAFs ranging from 70 to 85%) (Supplementary Table 4). In both tumor samples, we observed the loss of the wild-type allele, which is reflected in the high VAFs observed for the mutation. Therefore, based on these findings, we conclude that the model for CTCs determination is valid for all patients included in this study. To assess the impact of increased blood volumes on the detection of tumor components, ctDNA detection was examined in pre-treatment plasma samples from 9 patients using standard (5 mL) and higher volumes (20 or 40 mL). We detected ctDNA in all 9 (100%) plasma samples using 20 or 40 mL, while only 6/9 (66.66%) samples showed ctDNA using the conventional volume (Fig. 1c, Supplementary Table 5). For the conventional volume, the VAF threshold for mutation detection in our series was 0.07% (Supplementary Table 5). Notably, the observed VAFs of mutations using the manual extraction protocol with increased plasma volumes were significantly higher compared to conventional methods (Wilcoxon matched-pairs test, *n* = 12, *P* = 0.03) (Supplementary Fig. 4).

**Fig. 2:**
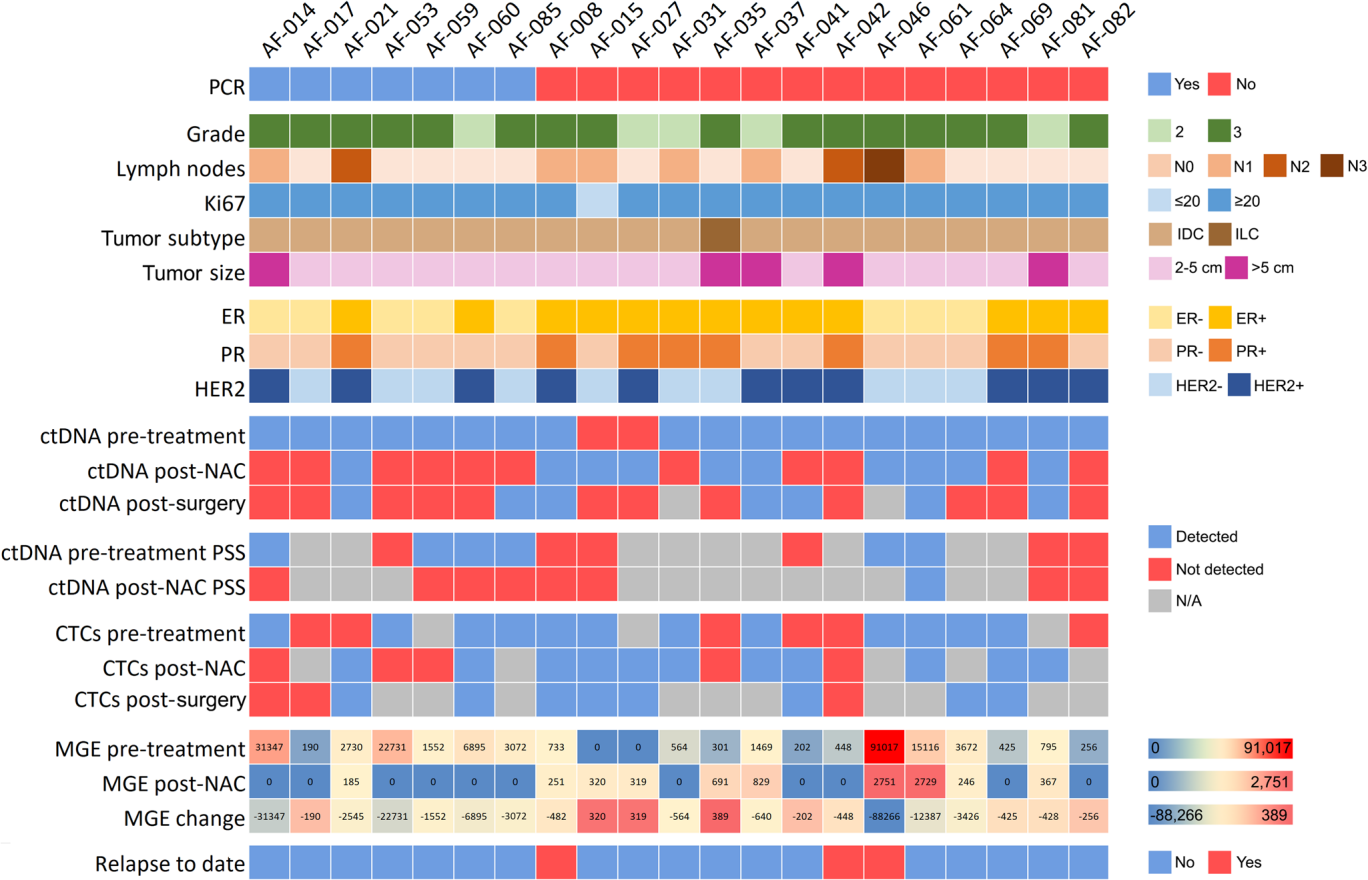
Summary of clinicopathological characteristics and ctDNA/CTCs detection using ddPCR and PSS technologies. The plot summarizes the presence/absence of ctDNA/CTCs and provides information on the inferred mutant genomic equivalents (MGE). The change in detection between pre- and post-neoadjuvant chemotherapy (NAC) plasma samples is indicated. Axillary lymph node status is categorized as follows: N0 (not affected), N1 (1–3 affected nodes), N2 (4–9 affected nodes), and N3 (10 affected nodes). The table also includes PCR results in surgical tissue and the relapse status. PCR pathological complete response, IDC invasive ductal carcinoma, ILC invasive lobular carcinoma, NAC neoadjuvant chemotherapy, PSS Plasma SeqSenseiTM, ER estrogenic receptor, PR progesterone receptor, ctDNA circulating tumor DNA, CTCs circulating tumor cells, MGE mutant genomic equivalents.

**Fig. 3:**
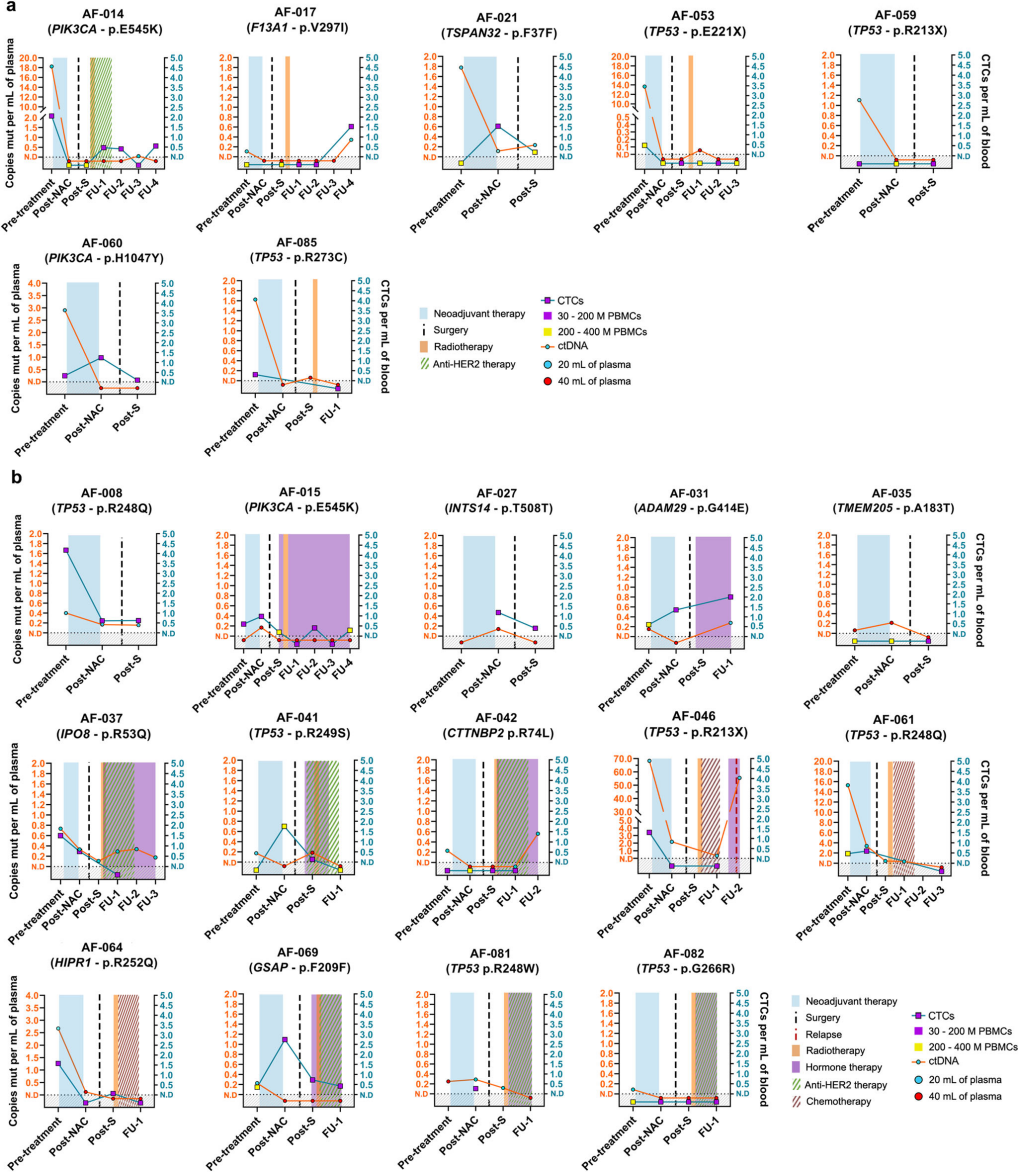
Disease monitoring using ctDNA/CTCs detection in pre- and post-treatment blood samples. Results for detection in pre-treatment, post-NAC, post-surgery, and during follow-up for (**a**) patients with PCR and (**b**) patients not reaching PCR in the tumor tissue. NAC neoadjuvant chemotherapy, Post S post-surgery, ctDNA circulating tumor DNA, CTCs circulating tumor cells, PBMCs Peripheral blood mononuclear cells.

Pre-treatment analysis involved 27 plasma samples (540 mL total volume) and 28 PBMCs samples using patient-specific ddPCR assays. The median detectable ctDNA VAF was 0.09% (range 0.01%–14.61%), with a median of 0.73 mutant copies per mL of plasma (range 0.06–68.3) (Fig. 1d, Supplementary Table 2). The minimum variant allele frequency (mVAF) for ctDNA was 0.01% at this stage. CtDNA was detected in 19/21 (90.47%) patients (Figs. 2, 3, Supplementary Table 2). CTCs detection had a lower limit of 0.30 CTCs per mL of blood, with a median of 0.60 CTCs per mL of blood (range 0.30–4.16 CTCs) (Fig. 1d, Supplementary Table 2). After patient exclusion (Fig. 1e), CTCs were detected in 12/19 (63.15%) samples (Figs. 2, 3). Notably, an association was observed between CTCs per mL of blood and lymph node involvement (Kolmogorov-Smirnov test, *n* = 18, *P* < 0.05) as well as between increased mutant copies per mL of plasma and triple-negative BC (TNBC) subtype (Supplementary Fig. 5). Combining both tumor components enabled disease detection in all 20/20 (100%) pre-treatment patients (Figs. 1e, 2, 3, Supplementary Table 2) using a median of 73.61 mL (20.37–203.78 mL) of blood. Additionally, ctDNA and CTCs were detected in a median of 8 (range 6–19) and 12 (range 8–27) reactions, respectively, in the ddPCR assays (Supplementary Table 2).

### Detection of RD in blood samples after NAC in early BC patients

Post-NAC, RD was assessed in 35 plasma samples (700 mL total volume) and 25 PBMCs samples from the cohort. ctDNA was detected in 10/21 (47.61%) patients with a mVAF of 0.007% and a median VAF of 0.015% (range 0.007%–0.238%) (Figs. 1D, 2, 3, Supplementary Table 2). The median mutant copies per mL of plasma was 0.191 (range 0.114–3.37) (Supplementary Table 2). Subsequent to excluding patients (Fig. 1e), CTCs were detected in 11/16 patients (68.75%) with a lower limit of 0.26 CTCs per mL of blood and a median of 1.17 CTCs per mL of blood (range 0.26–2.73) (Figs. 1c, 3, Supplementary Table 2). In the case of patient AF-059, the presence of FAM droplets in the germline controls led to the invalidation of positivity. However, ctDNA was detected in 3 of these 5 patients, indicating positive blood RD (Fig. 2). The combination of ctDNA and/or CTCs detected RD after NAC in 14/21 (66.66%) analyzed patients (Figs. 2, 3). DNA was partitioned in a median of 14 (range 8–20) and 12.5 (range 4–25) reactions for ctDNA and CTCs detection, respectively, in the ddPCR assays (Supplementary Table 2). Overall, a median of 65.78 mL (range 20.48–153.60 mL) of total blood was necessary for ctDNA and CTCs detection in this setting.

Overall, blood RD was detected in 12/13 patients (92.30%) and identified in 2/5 patients (40%) with PCR in the tumor tissue (Figs. 1e, 2, 3). Thus, this dual detection blood test exhibited 92.31% sensitivity (CI 95%: 63.97% to 99.81%) for detecting RD after NAC when the disease is present in the surgical tumor tissue.

**Fig. 4:**
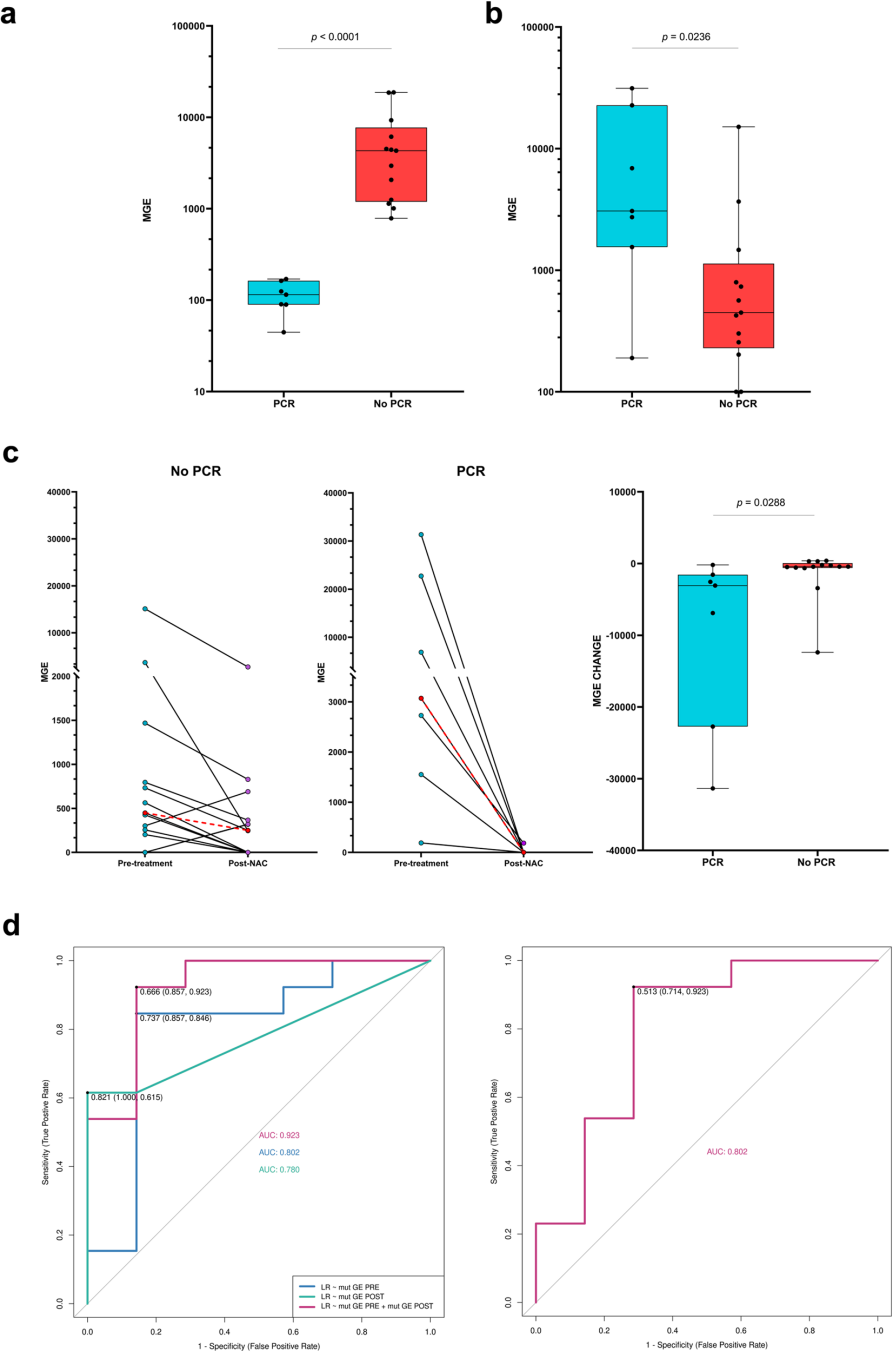
Dynamic ctDNA/CTCs analysis, tumor DNA detection in surgical tissue, and PCR prediction model. **a** Tumor DNA detection in surgical specimens. **b** ctDNA identification in pre-treatment plasma. **c** ctDNA dynamics during NAC, including MGE change. Red dots represent median. **d** ROC curves for NAC response prediction using MGE (left) and in silico cross validation (right). In the box-and-whiskers plots, the median is illustrated along with bars depicting the maximum and minimum values (*n* = 7 for PCR, *n* = 13 for no PCR). ctDNA circulating tumor DNA, CTCs circulating tumor cells, NAC neoadjuvant chemotherapy, PCR pathological complete response, MGE mutant genomic equivalents, ROC Receiver operating characteristic, AUC Area under the ROC Curve.

Using ddPCR assays, we assessed tumor cell presence in surgical specimens without microscopic detection of cancer cells (patients with PCR, *n* = 7), compared to samples where PCR was not reached. One patient’s tissue lacked tumor cells in the available block. A significant difference in median mutant genomic equivalents (MGE) was observed between tissues with and without PCR (median 114.90 in PCR versus 4312.66 in no PCR tissues, two-sided Mann–Whitney U test, *n* = 20, *P* < 0.0001) (Fig. 4a). No differences in tissue MGE were observed between PCR patients with positive and negative blood RD. However, the levels of MGE were statistically significantly higher in PCR patients with in-situ tumor sites compared to tissues with no visible tumor cells (Supplementary Fig. 6A,  B).

MGE in the pre-treatment blood sample and its evolution after NAC were assessed to predict PCR in the tumor. The patient AF-046 was excluded due to unique disease presentation with two primary breast tumors of different subtypes and more than 10 affected nodules (Fig. 2, Table 1). Importantly, this patient experienced a relapse and exhibited a significantly higher pre-treatment MGE compared to the median of the remaining samples (91,017 versus a median of 648). Furthermore, this patient showed one of the highest post-NAC MGE values (2751 versus a median of 167). Considering the remaining samples, pre-treatment plasma MGE levels were significantly higher in patients with PCR in the tumor compared to non-responders (two-sided Mann–Whitney U test, *n* = 20, *P* = 0.02) (Fig. 4b). In addition, responders showed a 6.85-times higher median MGE change before and after NAC compared to non-responders (median 448.24 for no PCR and 3072.48 for PCR in the tumor, two-sided Mann–Whitney U test, *n* = 20, *P* = 0.02) (Figs. 2, 4c). A prediction model for determining PCR in the tumor tissue was constructed using pre- and post-NAC plasma MGEs. Area under the ROC Curve (AUCs) of 0.80 and 0.78 were calculated when considering either pre- or post-treatment ctDNA levels, respectively (Fig. 4d). Considering both MGE levels in the model increased the AUC to 0.92 (Fig. 4d). In-silico cross-validation yielded an AUC of 0.80 (Fig. 4d) for PCR prediction.

### MRD detection in post-surgery blood samples from early BC patients

**Fig. 5:**
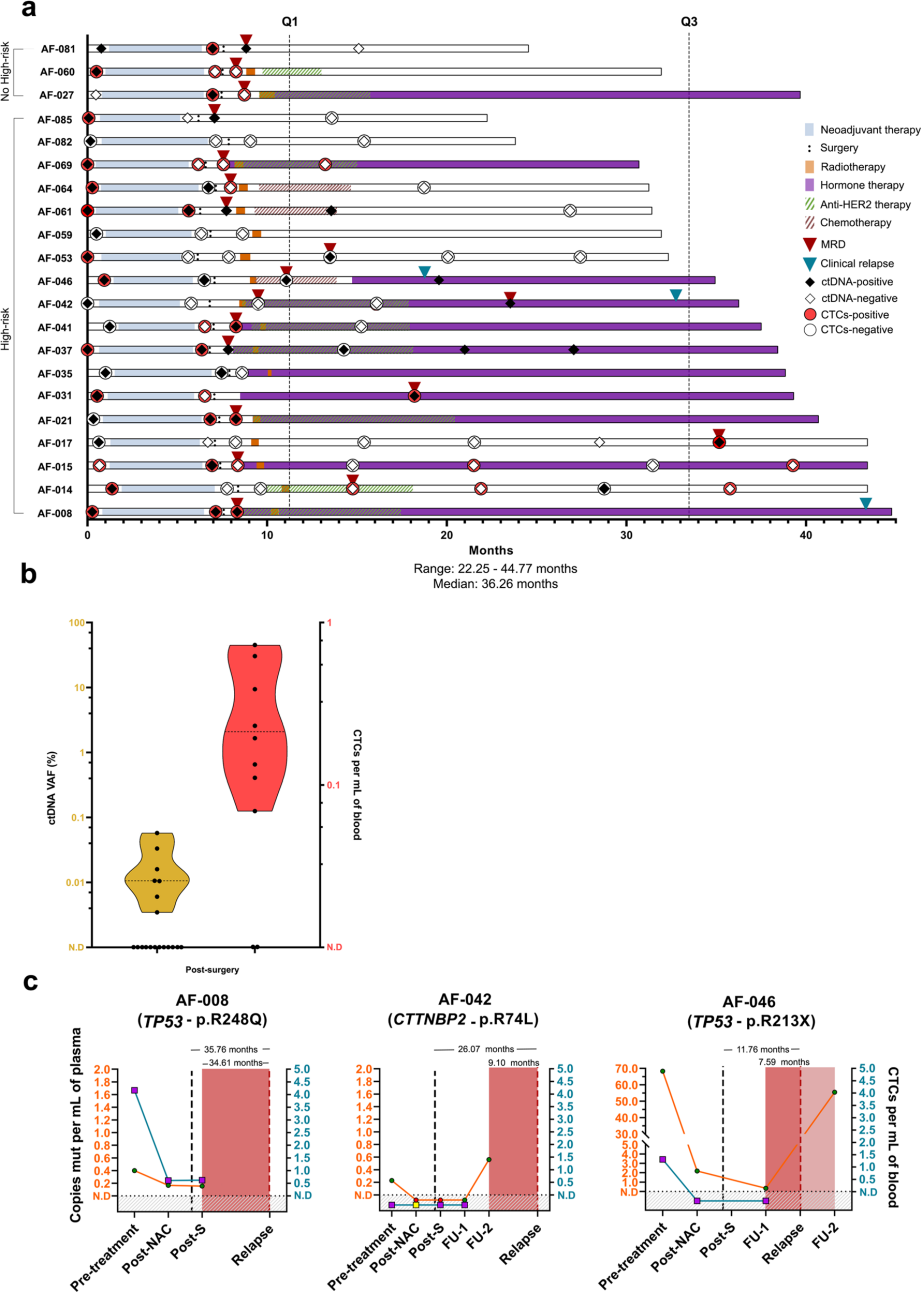
After surgery minimal residual disease (MRD) detection. **a** Swimmer plot illustrating the prospective clinical follow-up for the patients, indicating their treatments along with ctDNA/CTCs detection and clinical relapse status. **b** Detection of ctDNA (orange) and CTCs (red) in the post-surgery and follow-up blood samples (medians are illustrated as dashed lines and the upper and lower limits of the plots representing the maximum and minimum values, respectively). **c** Graphs show patients who experienced relapse, indicating the time in months from surgery to clinical relapse and MRD detection, as well as from MRD to clinical relapse. MRD minimal residual disease, ctDNA circulating tumor DNA, CTCs circulating tumor cells, NAC Neoadjuvant chemotherapy, FU Follow-up.

To identify MRD after surgery and anticipate relapse, we processed a total of 83 plasma samples (1660 mL) and 47 PBMCs samples derived from blood extractions collected 1 month after surgery and subsequently every 6 months from high-risk patients (*n* = 18) (Fig. 1a). To date, the median clinical follow-up was 36.26 months (Fig. 5a), with multiple blood extractions conducted during surveillance (Supplementary Table 2).

Post-surgery, ctDNA was detected in 7/19 patients (36.48%), after patient exclusion due to sample unavailability (Figs. 1e, 2, 3, Supplementary Table 2). The mVAF was 0.003%, the lowest VAF observed for ctDNA detection in all tested samples (30 ppm of mutant DNA copies) (Figs. 3, 5b, Supplementary Table 2). The median VAF for ctDNA detection was 0.011% (range 0.003%–0.057%), with a median of 0.156 mutant copies per mL of plasma (range 0.06–0.432) (Figs. 3, 5b, Supplementary Table 2). Subsequent to the exclusion of patients because lack of sample (Fig. 1e), CTCs positivity was observed in 8/10 patients (80%) with a median detection of 0.21 CTCs per mL of blood (range 0.06–0.72 CTCs) (Figs. 3, 5b, Supplementary Table 2). The lower limit of detection for CTCs was 0.06 CTCs per mL of blood, the lowest among all analyzed samples. After excluding patients due to a lack of sample (Fig. 1e), when evaluating MRD detection at the post-surgery stage, positive results for ctDNA and/or CTCs, including assessments with no repetitions, identified 12 out of 19 (63.15%) blood samples as MRD positive (Fig. 3, Supplementary Table 2).

In subsequent follow-up blood samples only from high-risk patients, ctDNA was detected with a median VAF of 0.022% (range 0.003–0.061%) (Fig. 3, Supplementary Table 2). A median of 0.315 mutant copies per mL of plasma (range 0.054–0.560) was measured (Supplementary Table 2). For CTCs, a median of 0.445 CTCs per mL of blood (range 0.289–2.002) was observed (Fig. 3, Supplementary Table 2). Considering MRD positivity as ctDNA and/or CTCs detection, in tumor tissue PCR patients with samples at follow-up, two of them (AF-053 and AF-085) showed a transformation from MRD detection into a negative status after post-surgery treatment, while one patient (AF-017) tested positive in her last follow-up both for CTCs and ctDNA detection, and one (AF-014) did not show negativity in any of the follow-up timepoints (Fig. 3, Supplementary Table 2). Among patients without PCR, one (AF-082) consistently tested negative in all timepoints after surgery, while four (AF-041, AF-061, AF-064, AF-081) transformed into MRD-negative status during adjuvant therapy. In contrast, five patients were MRD-positive at some point throughout the follow-up period (AF-015, AF-031, AF-037, AF-046, AF-069). To date, three patients have clinically relapsed with metastatic disease. Patient AF-008, who did not undergo blood follow-up due to non-adherence to our high-risk criteria, experienced a relapse 35.76 months after surgery and exhibited positive blood MRD in the post-surgical sample (1.15 months after surgery). At her last follow-up timepoint, patient AF-042 converted to MRD positivity. She subsequently relapsed 26.07 months after surgery, and blood MRD positivity was detected 16.96 months post-surgery. Another patient, AF-046, experienced a clinical relapse 11.76 months after surgery with MRD positivity detected 4.17 months post-surgery at the ctDNA level. MRD detection in blood preceded the clinical relapse by 34.61, 9.10, and 7.59 months in AF-008, AF-042, and AF-046 respectively (Fig. 5a). The VAFs ranged from 0.019% to 0.06% for ctDNA detection and 0.62 CTCs per mL of blood in these patients. It is noteworthy that patient AF-008 exhibited the highest pre-treatment CTCs levels with triple metastatic sites in the lung, bone, and liver, and patient AF-046 presented the highest pre-treatment ctDNA levels among all patients in this cohort. In addition, patient AF-042 developed a central nervous system metastasis. It is also worth mentioning that, at the time of manuscript acceptance, the remaining patients in the study are currently free of disease.

To enable ultrasensitive detection of ctDNA and CTCs at the post-surgery and follow-up time points, the DNA was partitioned into a median of 15 reactions (range 26–4) and 16 reactions (range 8–32), respectively, in the ddPCR experiments. A median of 94.87 mL of total blood was required to facilitate ultrasensitive MRD detection.

Importantly, this study employs stringent control measures to control false positive events by incorporating a substantial number of negative controls (Fig. 1a, b). Furthermore, we conducted a comparison between the calculated maximum sensitivity, accounting for the DNA input in each experiment, and the observed VAF for ctDNA detection. It is worth noting that the maximum sensitivity is significantly lower than the observed mutant VAFs in the samples, thus emphasizing the robustness of our methodology (Supplementary Fig. 7 and Supplementary Table 2).

### Orthogonal validation of ctDNA detection utilizing an ultrasensitive NGS panel (Plasma-SeqSensei^TM^)

Detection of ctDNA was validated in a subgroup of 37 pre-NAC, post-NAC, post-surgery, and follow-up plasma samples with a fixed NGS panel using unique molecular identifiers (UID) for ultra-sensitive tumor DNA detection. Comparable plasma volumes were employed for these samples from 12 patients, with 5 achieving PCR and 7 showing PCR negativity in the tumor tissue (Supplementary Fig. 8, Supplementary Table 6). Among the 12 pre-treatment plasma samples, 6 (50.00%) tested positive for ctDNA using both ddPCR and NGS panel for the selected mutation (Supplementary Fig. 8, Supplementary Table 6). ddPCR detected ctDNA in 5/12 (41.67%) where the NGS panel yielded negative results in all cases (Supplementary Fig. 8, Supplementary Table 6). One sample (AF-015) tested negative with both approaches (Supplementary Fig. 8, Supplementary Table 6). In the post-NAC samples, ctDNA was negative in all NGS panel-tested samples except one (AF-061), which also showed positivity in ddPCR assays. Conversely, ddPCR detected ctDNA in 4/9 (44.44%) samples for the selected mutation (Supplementary Fig. 8, Supplementary Table 6).

At post-surgery and follow-up time points, we analyzed 16 samples using both the PSS panel and ddPCR technologies for ctDNA detection. Interestingly, the PSS panel yielded negative results for ctDNA detection in all samples, while ddPCR demonstrated positive results in 8/16 (50%) samples (Supplementary Fig. 8, Supplementary Table 6).

Overall, there was a strong correlation between ddPCR and NGS panel for samples positive with both technologies (Supplementary Fig. 9). The NGS panel detected mutations that were not analyzed with ddPCR (Supplementary Table 6). Additionally, through the design and implementation of specific assays for the corresponding time point germline DNA, two mutations were identified as CHIP events by ddPCR (Supplementary Table 6).

## Discussion

In this study, we have developed a liquid biopsy method to detect RD and MRD in BC patients by increasing the volume of blood drawn. Our findings demonstrate an improved detection of ctDNA and CTCs by using larger plasma volumes. Furthermore, we noted enhanced detection of ctDNA with higher VAFs through manual extraction methods^25^ compared to column-based approaches, likely attributed to superior cfDNA quality and reduced germline DNA contamination (Supplementary Fig. 1). In this context, we observed that high plasma volumes detected ctDNA in all samples, whereas conventional volumes only detected ctDNA in 66.66% of the samples, as shown in previous studies^16–19^.

In pre-treatment plasma samples using a maximum of 40 mL, we achieved a mVAF detection of 0.01% and a median of 0.73 mutant copies per mL of plasma, representing a significant improvement over previous studies using conventional plasma volumes^17^. Our approach exhibited one order of magnitude higher sensitivity and a comparable VAF median to the most sensitive NGS approach for a similar patient population^13^. We detected ctDNA in 90.47% of the samples. This detection percentage is superior to previous studies^14,17,18,26,27^. In addition to ctDNA, we also investigated the detection of CTCs using ddPCR in pre-treatment blood samples. Our study represented the first application of ddPCR for CTCs detection in blood. We observed a CTCs detection percentage of 63.15%, which markedly increased the sensitivity compared to previous studies that reported a detection rate of 20–25%^28–30^. Furthermore, for positive samples, we identified a median CTCs count of 0.60 CTCs per mL of blood, consistent with previous investigations^31^. Importantly, we observed an association between pre-treatment CTC count per mL of blood and affected lymph nodes, which is also in line with previous studies^32^. We also observed elevated levels of ctDNA in TNBC tumors, which aligns with the higher proliferation rates and increased shedding of ctDNA typically associated with this tumor type^33^. Overall, we detected tumor components in 100% of the patients in this setting similar to previous investigations using patient-specific NGS panels and intensive sequencing processes^13^.

After NAC treatment, we observed low levels of ctDNA in plasma (median VAF: 0.015%, mVAF: 0.007%), which is comparable to the most sensitive study in a similar patient population^13^. Additionally, CTCs were detected in a high percentage of blood samples (68.75%), consistent with previous investigations where CTCs were found even at early stages of BC^29,30,32,34–37^. As we observed, previous studies have also reported no tumor tissue PCR prediction based on CTCs counts after NAC^29,30,38^. Overall, blood RD was detected in 33.33% of patients who achieved PCR, an inferior percentage compared to a previous study^13^. Therefore, we observed an improved specificity and may suggest the presence of distant micrometastasis or incomplete clearance of tumor components at the time of sample extraction. In patients not achieving PCR, we detected blood RD in 92.30%, also similar to previous reports with patient-specific NGS panels^13,16^.

In the validation of ctDNA detection using the PSS commercial NGS panel, pre-treatment samples exhibited 50% ctDNA positivity with both methods. However, ddPCR demonstrated higher sensitivity, detecting ctDNA in samples that NGS missed. After NAC, ddPCR detected ctDNA in 44.44% of samples, while NGS mainly yielded negative results. Similarly, post-surgery, ddPCR identified more ctDNA in plasma samples than the PSS panel. Notably, samples with negative ctDNA detection using NGS had ddPCR levels below the NGS panel’s limit of detection (VAF of 0.06%). Our findings emphasize the importance of using germline DNA control to manage CHIP events in NGS assays.

In the surgical specimen, RD-positive patients achieving PCR in the tumor tissue exhibited similar tumor DNA levels as other PCR cases. We observed that ddPCR clearly stratified tumors with and without PCR, offering an alternative to visual tissue examination. Furthermore, the PCR tumor tissues with observable in-situ tumor sites presented significantly elevated tumor DNA levels, as expected. We hypothesize that the presence of macrophages and apoptotic cells may contribute to the detection of minute amounts of mutant DNA in tissues with non-visible tumor cells, as assessed by the pathologist.

We next evaluated the association of total plasma MGE levels and their dynamics with NAC outcome. At pre-treatment, we observed that MGE were statistically significantly higher in patients with PCR compared to those without PCR. Importantly, the relapsed patient displayed higher MGE levels in both pre- and post-treatment plasma samples compared to the rest of the samples. In addition, analyzing the dynamics of MGE between pre- and post-treatment ctDNA revealed that patients with ctDNA clearance at the end of NAC were more likely to reach PCR in the tumor tissue as was previously shown^13,16^. Importantly, we developed a model (AUC = 0.92) combining both pre- and post-treatment ctDNA levels but also employing only pre-treatment ctDNA levels to predict NAC response (AUC = 0.80). This result improves previous investigations where only the ctDNA level after NAC was capable of predicting PCR^13^.

At the post-surgery stage, we observed the lowest ctDNA VAF with an mVAF of 0.003%. It is noteworthy that this time point typically corresponds to minimal disease burden. This level of sensitivity has only been demonstrated in post-NAC plasma samples by the most sensitive study to date^13^. Additionally, we observed the lowest numbers of CTCs per mL of blood in the post-surgery samples. These findings suggest that the utilization of our methodology for MRD detection is likely to enhance the anticipation of clinical recurrence compared to previous studies in early BC^16,17,39^. However, clinical surveillance is necessary to draw definitive conclusions about the clinical significance of our findings^16,17,40,41^.

Herein, we employed an NGS approach—the Plasma-SeqSensei technology—for ultrasensitive detection of ctDNA and utilized it as an orthogonal validation method. With a claimed LOD of 0.06% in VAF, this NGS panel was chosen for the first time in our research. While we validated the high levels of sensitivity claimed by the manufacturer, we also observed that our methodology exceeded this threshold, demonstrating its capability to detect ctDNA at even lower VAFs.

In this study, we included a cohort of high-risk disease patients, extending our sample collection even into the post-surgery follow-up for those at the highest risk. Our results have identified four patient groups: (i) those consistently MRD-negative in all follow-up blood samples (AF-082), (ii) a subgroup not consistently negative during follow-up (AF-014, AF-015, AF-031, AF-037, AF-046, AF-069), (iii) a subgroup achieving negativity during or after treatment (AF-041, AF-053, AF-061, AF-064, AF-081, AF-085), and (iv) individuals initially testing negative but later turning positive during follow-up (AF-017, AF-042). The third group of patients underscores the pivotal role of adjuvant chemotherapy in enhancing clinical outcomes in early BC management^42^. Conclusions pertaining to the rest group of patients will be attainable as we extend the clinical follow-up period. It is noteworthy that we included an increasing number of HR-positive patients in our cohort who tend to experience relapses beyond the time frame covered by this study’s clinical follow-up^43,44^. Notably, our methodology successfully predicted the recurrence of three patients after surgery, 34.61, 9.10, and 7.59 months earlier than clinical evidence. In patients AF-008 and AF-046, we detected blood RD as ctDNA, with a VAF of 0.05% (0.15 mutant copies per mL) and 0.06% (0.34 mutant copies per mL). This level of detection was previously unattainable using conventional plasma volumes^17^. Therefore, our results not only build upon previous investigations but also validate the efficacy of our assay for blood MRD detection. Of note, patient AF-008 exhibited the highest levels of CTCs and presented with multiple metastatic sites. This suggests a highly disseminated disease with metastatic potential, even at early tumor stages. Additionally, patient AF-042 had metastasis in the central nervous system, which is challenging to detect in blood^17,18,45^ and may explain the more delayed identification of blood MRD in relation to her clinical relapse.

In this study, high-throughput sequencing detected somatic mutations in tumor tissues, with *TP53* and *PIK3CA* genes having the highest mutation frequency (52.38%). Therefore, more restricted targeted NGS panels can also effectively identify mutations to be tracked by ddPCR. In this context, considering a previously published fixed breast cancer gene panel that demands fewer sequencing resources^46^, and discarding *TP53* and *PIK3CA* mutations already present in the panel, we found that all patients’ tumors exhibited somatic mutations suitable for tracking in their blood samples using the proposed methodology (Supplementary Fig. 10). Furthermore, we show that alternative sequencing technologies, such as RNAseq, could also be utilized for the detection and tracking of truncal somatic mutations using this methodology.

Significant efforts have been invested in optimizing sequencing technologies to enhance ctDNA detection sensitivity. Various companies have developed strategies, like patient-specific mutation panels, for clinical investigations^33,47–49^. These technologies have shown successful applications in detecting MRD in early BC. They represent robust methodologies adhering to rigorous quality standards. However, despite their efficacy and exceptional sensitivity, they have yet to receive regulatory approval for clinical use in this context. Therefore, further evidence regarding their clinical utility is required before considering widespread adoption in clinical practice. Moreover, the significant cost per sample imposed by commercial entities and issues related to sequencing quality present barriers to their integration into routine clinical workflows, particularly in lower-income countries^50^.

Our study introduces a novel methodology that significantly advances the landscape of cancer detection and monitoring. One of the key innovations lies in our approach to ctDNA detection, where we leverage higher plasma volumes. This strategy not only enhances the sensitivity of ctDNA detection but also offers a pragmatic and easily implementable solution for identifying extremely localized cancers. Furthermore, our study achieves a milestone in the field by concurrently detecting both CTCs and ctDNA using a single blood sample, the easily implementable ddPCR platform, and one truncal point somatic variation. This study differentiates from previous investigations using common blood draws that necessitated the use of whole-genome sequencing to detect structural variations, which are more difficult to identify, and designed PCR assays to be tracked in the blood of the patients in a clinical context^18^. Notably, our study marks the first instance of employing a ctDNA and CTCs detection approach in both pre- and post-surgery blood samples, with the primary goal of detecting residual disease after NAC and relapse before it becomes clinically evident. This ultrasensitive dual detection capability provides a comprehensive and holistic assessment of circulating tumor components, offering a more nuanced understanding of the disease’s dynamics.

Moreover, our study enhances detection capacities by utilizing highly partitioned ddPCR, significantly improving sensitivity and bolstering the overall robustness of our approach. In doing so, we have effectively integrated capabilities from other technologies that are no longer available. This integration involves increasing the number of droplets, thereby enhancing the separation and detection of DNA molecules, promoting mutant DNA detection^51–53^.

The methodology presented herein achieves similar sensitivity to the best NGS-based techniques by targeting a single truncal somatic mutation. Additionally, it enables CTCs detection and offers a more comprehensive understanding of the disease’s dynamics. While the proposed methodology requires larger blood volumes and manual plasma DNA extraction, it is crucial to highlight the minimal treatment history of these early breast cancer patients, who generally exhibit favorable health conditions. Additionally, the cfDNA manual extraction method is easily implementable in a laboratory setting and is cost-effective. Therefore, the clinical accessibility and potential impact on disease progression outweigh any inconveniences. In conclusion, our study presents a robust methodology involving large plasma volumes, highly partitioned ddPCR assays, and rigorous controls. Further validation with larger cohorts is necessary, but our findings present a promising tool for clinical studies with a focus on de-escalating NAC, avoiding surgery when blood RD is negative, or monitoring responses to adjuvant treatments.

## Methods

### Study design

This prospective observational study aimed to develop a methodology for detecting ctDNA and CTCs in early BC patients before and after treatment. Blood samples were collected from January 2020 from localized or locally advanced BC patients scheduled to receive NAC. Prior to sample collection, all participants provided written informed consent, and the study obtained the corresponding permission from the ethics committee (“Servicio Andaluz de Salud (SAS) – Consejería de Salud”). We have complied with all relevant ethical regulations including the Declaration of Helsinki. We affirm that none of the patients experienced any adverse effects resulting from their blood donation to this study. The response endpoint was PCR in the tumor tissue after NAC, defined as the absence of residual invasive tumor cells in the breast and lymph nodes at the time of surgery. Clinical relapse was defined as tumor detection by computed tomography scan.

### Patients and samples

Twenty-one patients with localized or locally advanced BC underwent standard treatment with NAC with or without trastuzumab, depending on their HER2 status. Patients with distant metastatic disease were excluded. Clinicopathological characteristics of the patients are presented in Table 1.

Pre-treatment tumor biopsies were extracted using core needle biopsies and frozen in RNA later (Sigma-Aldrich) to extract both DNA and RNA. Immunohistochemical (IHC) analysis was performed in a formalin-fixed paraffin-embedded (FFPE) core needle biopsy to quantify expression of human epidermal growth factor receptor 2 (HER2), hormone receptors (HR), and Ki67. Estrogenic receptor (ER) and progesterone receptor (PR) were considered positive in tumors presenting more than 1% nuclear-stained cells. HER2 staining was scored according to the guidelines^54^. HER2 status was considered positive when graded as 3+, while 0 to 1+ were negative and 2+ was an inconclusive result and silver in situ hybridization was performed.

Blood samples were collected in citrate blood bags and processed within 2 h following venipuncture. Serial blood samples were extracted before any treatment, after NAC, one month after surgery, and in 6 months´ intervals during follow-up for those patients presenting high risk-of-relapse disease defined as: grade 3 and/or affected axillary lymph nodes and/or HER2-positive/TNBC tumors. Blood samples collected at each timepoint were systematically processed and subdivided into 20 mL plasma tubes and 100 million blood cell tubes to enable the determinations outlined in this study. The total amount of blood required for ctDNA and/or CTCs detection per timepoint is detailed in Supplementary Table 2.

### Tumor tissue DNA/RNA and buffy coat DNA extraction

Pre-treatment tumor tissues were stained with hematoxylin-eosin and marked for tumor content by a qualified pathologist to achieve >40% in tumor cells in the macro-dissected area. Tumor tissue DNA and RNA was extracted using the RecoverAll™ Total Nucleic Acid Isolation Kit (ThermoFisher Scientific).

Germline DNA was extracted from the discarded blood cells, obtained from the negative immunoselection process to enrich for CTCs in the pre-treatment blood samples. The DNA was extracted using the QIAamp DNA Blood Mini Kit (Qiagen). This DNA was used as germline control for each patient/timepoint to check for false positives and clonal hematopoiesis of indeterminate potential (CHIP) in the assays both for ctDNA and CTCs detection.

DNA quantification was performed using RNAseP ddPCR assay (Bio-Rad) as previously described^17^. RNA was quantified in the Nanodrop One platform (ThermoFisher scientific).

### Plasma DNA extraction

Plasma DNA was extracted using a bead-based scalable methodology as previously described adapted to 20 mL of plasma (solid version)^25^. In addition, a subset of plasma samples was extracted using QIAamp Circulating Nucleic Acid Kit (Qiagen) to compare cfDNA quality between methodologies. Plasma DNA was quantified using RNAseP ddPCR assay (Bio-Rad).

See Fig. 1 for details about the methodology workflow to detect ctDNA.

### CTCs/MFC7 cells enrichment and DNA extraction

Peripheral blood mononuclear cells (PBMCs) were obtained by centrifugation of buffy coat in Ficoll gradient according to manufacturer’s instructions, and vitally frozen following the extraction. PMBCs were enriched in CTCs/MFC7 cells using the EasySep Human CD45+ Depletion Kit II (StemCell technologies). As above-mentioned, DNA from normal blood cells was used as germline controls. Total cell counts were assessed immediately after thawing and before enrichment. PMBCs were enriched in CTCs/MFC7 cells using either 1 or 2 steps of the EasySep Human CD45+ Depletion Kit II (StemCell technologies), which consists of immunomagnetic negative selection to remove blood lymphocytes. A median depletion efficiency of 3.48(log) was calculated, which closely aligns with the manufacturer’s specifications of 4.0 (log) (Supplementary Table 2). Then, total DNA was extracted using the QIAamp DNA Blood Mini Kit (Qiagen) and quantified using RNAseP ddPCR assay (Bio-Rad).

A model using MFC7 cells to estimate CTCs/mL of blood was performed in experimental triplicates.

See Fig. 1 for details about the methodology workflow to detect CTCs.

### Whole-exome sequencing

Both tumor and germline DNA from 19 patients was subjected to whole exome sequencing (WES) using the Agilent V6 exome kit (Agilent-BGI genomics) and a DNB-seq sequencer. Seventy to 810 ng of tumor DNA and 760 to 1000 ng of germline DNA were employed to construct WES libraries. Samples were tested for integrity and purity and fragmented using the Covaris system. Then, 150 to 250 bp fragments were selected from the fragmented genome using magnetic beads. The fragments were subjected to end-repair, 3’ adenylation, and adapter ligation. The selected fragments were amplified and hybridized with probes capturing the whole exome. The captured fragments were amplified and circularized to be sequenced.

Quality control of WES data was performed using fastQC (v0.11.9) followed by paired-end reads adapters trimming and quality filtering using Trim Galore (v0.6.7). Pre-processed reads were mapped to the GRCh38 reference genome by BWA-mem (v0.7.17). Data correction for technical biases and somatic mutation calling were performed according to GATK’s best practices (https://software.broadinstitute.org/gatk/best-practices). The resulting aligned SAM files were sorted by coordinate using Picard Sortsam (Picard v2.26.10) and converted to BAM format with Samtools (v1.9). Picard MarkDuplicates was run to mark duplicated reads from each BAM file and GATK BaseRecalibrator and ApplyBQSR (GATK v4.2.2.0) were used for base quality score recalibration. Somatic variants analysis for each tumor sample was performed by GATK Mutect2 in matched normal mode including a custom panel of normal non-cancer variations which was previously built and a germline variant annotation file for the GRCh38 reference genome obtained from the GATK resource bundle. Reads counting summaries were obtained for tumor and normal samples using GATK GetPileupSummaries and passed to GATK CaculateContamination for contamination calculation. Finally, reported variants were filtered to get true somatic mutations using FilterMutectCalls. Somatic variants were annotated by ANNOVAR (v20200608) with custom made databases for COSMIC v95 and TCGA mutation data retrieved from GDC data portal^55^.

### Whole-transcriptome sequencing

In the surgical specimens from the patients AF-008 and AF-014 whole transcriptome sequencing (RNAseq) was performed to detect somatic variants. Tumor RNA was subjected to the whole transcriptome sequencing via rRNA depletion (Genewiz company). RNAseq libraries were constructed using a ribosomal RNA depletion step, following fragmentation and random priming, cDNA synthesis, end-repair, 5’ phosphorylation, dA-tailing, adaptor ligation, and PCR enrichment. Sequencing was performed on an Illumina NovaSeq, PE 2 × 150.

Sequence reads were trimmed to remove adapter sequences and nucleotides with poor quality using Trimmomatic v.0.36. The trimmed reads were mapped to the Homo sapiens GRCh38 reference genome available on ENSEMBL using the STAR aligner v.2.5.2b. BAM files were generated as a result of this step.

A SNP/INDEL (Single Nucleotide Polymorphism / Insertion or Deletion) analysis was performed using mpileup within the Samtools v.1.3.1 program followed by VarScan v.2.3.9. The parameters for variant calling were: minimum frequency of 25%, *p* value less than 0.05, minimum coverage of 10, minimum read count of 7.

### Copy number variation assessment

Two tumor samples (AF-059 and AF-085) with elevated mutation variant allele frequency (VAF) were selected to assess possible copy gains that could affect the extrapolation using the MCF7 model. CNVkit (v0.9.10) was employed to evaluate copy numbers for genomic regions containing mutations that were initially selected from WES normal-tumor paired variant calling analysis and subsequently validated by ddPCR. CNVkit was run in batch mode using GRCh38 reference genome and matched Ensembl gene annotation database to obtain log2 ratios for each genomic segment in the tumor-normal pairs. Copy number imputation was carried out using the clonal method of CNVkit’s call mode, taking into account the log2 ratio and adjusting for a diploid genome and tumor purity.

### ddPCR assays: assays validation and performance

ddPCR assays were designed and ordered using the algorithm included in the Custom TaqMan® SNP Genotyping Assays (ThermoFisher Scientific) ordering system. To select somatic variants and design ddPCR assays, we applied the following criteria: variants had to be annotated in COSMIC and in TCGA databases and they had to have the highest VAF in a given sample. When mutations in the genes *PIK3CA* or *TP53* were present, they were selected as they are well-known truncal somatic mutations in BC.

Specific ddPCR assays were validated using tumor and germline DNA from the corresponding patient. We considered an assay to be validated if we detected the mutation at an allele frequency similar to the one observed in WES for a given sample. Validated ddPCR assays were further optimized to identify annealing temperatures giving the best separation between mutant and wild type droplets. See Supplementary Table 7 for assay conditions.

Digital PCR was performed on a QX-200 ddPCR system (Bio-Rad) with primers and probes at a final concentration of 1X. PCR reactions were prepared with ddPCR Supermix for probes (Bio-Rad) and partitioned into droplets in the Auto droplet generator (Bio-Rad) according to manufacturer’s instructions. PCR reactions were run on 96 well plates using the C1000 Touch™ thermal cycler (Bio-Rad) incubating the plates at 95 °C for 10 min followed by 40 cycles of 94 °C for 30 s and specific assay extension temperature for 60 s, followed by 10 min incubation at 98 °C. The temperature ramp increment was 2 °C/sec for all steps. Plates were read on the Bio-Rad QX-200 droplet reader using QuantaSoft v1.7 software (Bio-Rad) to detect positive droplets for mutant DNA, wild type DNA, both or neither. Samples were partitioned based on the amount of DNA both for ctDNA and CTCs detection (Supplementary Table 2).

Importantly, to control for false positives and CHIP events, the same amount of the corresponding germline DNA was equally distributed in the same number of partitions for each assayed timepoint both for ctDNA and CTCs detection. Moreover, at least two negative control wells per sample with no DNA were included. Furthermore, to control false positivity, we specifically selected time points that exhibited 2 or 3 FAM droplets and only double positives (FAM and VIC). To ensure greater accuracy, we conducted 2 additional negative controls with germline DNA from the corresponding patient following the previously mentioned methodology (Fig. 1a, b, Supplementary Table 8).

### ddPCR assays: data analysis

A sample was considered as positive for ctDNA or CTCs if two or more FAM positive droplets (mutant allele) were detected in a replicate (we considered 20 mL of plasma or 200 million of blood cells as replicate). Samples with two droplets but detected in two separate replicates were considered negative. When a replicate was initially negative, and the second replicate was positive, wells from both replicates were considered to calculate mutant copies per eluate.

Control samples with no DNA template and negative control samples with the corresponding germline DNA were considered positive or negative following the above-mentioned criteria. If we detected any positivity in any of the controls, that replicate was considered inconclusive for the given time-point.

#### ctDNA

To calculate mutant copies per mL of plasma in each timepoint, the mutant copies per microliter (as obtained from the ddPCR platform (Bio-Rad)) were transformed into mutant copies per eluate:

1 $$\begin{array}{l}Mutant\,copies\,per\,eluate=Mutant\,copies\,per\,microliter\,x\,20\,microliters\\\qquad\qquad\qquad\qquad\qquad\qquad \,x\,number\,of\,wells\end{array}$$

Mutant copies per mL of plasma were calculated as follows:

2 $$Mutant\,copies\,per\,mL\,of\,plasma=\frac{Mutant\,copies\,per\,eluate}{mL\,of\,plasma\,employed}$$

For each timepoint, ctDNA VAF was transformed into MGE as follows:

3 $$MGE=ng\,cfDNA\,x\frac{1000\,pg}{3.3\,pg\,per\,haploid\,GE}x\,Variant\,allele\,frequency(VAF)$$

#### CTCs

To calculate the total number of CTCs, we used a spike-in experiment. Spike-in samples were generated combining 64 or 128 MFC7 cells with 200 million PBMCs from a healthy individual. Then, enrichment was performed as previously specified for patients´ samples and ddPCR was applied to detect the mutation E545K in the *PIK3CA* gene present in this cell line (https://cancer.sanger.ac.uk/cosmic/sample/overview?id=1998454). The experiments were performed in triplicate. Next, linear regression was used to generate a formula to obtain number of CTCs per sample.

4 $$Total\,number\,of\,CTCs=\frac{Mutant\,copies\,per\,eluate-0.3072}{0.1992}$$

In case of patient sample´s CTCs, CTCs per mL of blood was inferred from the spike-in experiment data as follows:

5 $$CTCs\,per\,mL\,of\,blood=\frac{Total\,number\,of\,CTCs}{mL\,of\,blood}$$

### Plasma-SeqSensei™ sequencing

The commercial NGS panel Plasma-SeqSensei^TM^ (PSS) (Sysmex) includes regions from the *AKT1*, *ERBB2*, *ESR1*, *KRAS*, *PIK3CA*, and *TP53* genes. For detailed information about the regions covered in the panel, please refer to Supplementary Table 9. We prepared sequencing libraries following the manufacturer’s instructions and used an Illumina NextSeq500 system for sequencing. Data analysis was performed using the provided manufacturer’s software.

### Statistical analysis

Differences in MGE in tissues with and without PCR, tumor tissues with in-situ tumor cells compared with the rest PCR tissues, MGE between PCR and non-PCR patients at pre-treatment, MGE change between pre- and post-NAC in PCR and non-PCR patients, CTCs per mL of blood in lymph node-negative and positive patients and ctDNA levels depending on tumor subtype were evaluated using non-parametric Mann–Whitney U and Kolmogórov–Smirnov tests and *t* test in GraphPad Prism v8.0.1. Differences VAF between conventional methodologies and the proposed workflows were assessed using Wilcoxon matched-pairs test. All *p* values were two-sided and considered significant at 0.05.

Linear regression analysis to compare ctDNA detection values between ddPCR and PSS was performed using GraphPad Prism v8.0.1.

Sensitivity for tumor tissue PCR prediction using ctDNA and/or CTCs at the post-NAC timepoint were calculated using the MedCalc online software (https://www.medcalc.org/calc/diagnostic_test.php).

To predict PCR in the tumor tissue based on clinical and genomic data, multivariable logistic regression models were developed using caret (v6.0.93), car (v3.1.1), and pROC (v1.18.0) R packages (https://www.R-project.org/). Two distinct approaches were employed for model creation: manual model creation and automated model creation. For the manual model creation approach, predictor variables were filtered based on their correlation with the target variable, and only those predictors which maximized Cramér’s V measure were kept to build the model. The relevance of each variable in the model was assessed by means of pseudo-R² coefficient and the variables with the greatest impact were included. For the automated model creation approach, multiple models were built on the basis of different automated variable selection methods (forward, reverse, and stepwise) using the Akaike Information Criterion (AIC) as models fit comparison criterion. The receiver operating characteristic (ROC) curve was used as a performance measure among all candidate models and the one maximizing the AUC was chosen as the final predictive model. DeLong’s test was used to test for AUC’s model significance in comparison to a random non-fitted model (AUC = 0.5) setting a significance level of α = 0.05. The chosen model was validated through a Leave-One-Out Cross Validation (LOOCV) process for internal testing and the resulting AUC was used as a measure of the model robustness. To obtain the specificity and sensitivity of the model, the probability cut-off point was chosen as the probability value that maximized the Youden index.

The statistical tests comparing the calculated maximum sensitivity to the observed Variant Allele Frequencies (VAFs) were conducted. Normality of the data was assessed using the Shapiro test, followed by Wilcoxon matched-pairs test (n ctDNA = 44, n CTCs = 37). All p-values were two-sided and considered significant at 0.05.

### Supplementary information

SUPPLEMENTARY INFORMATION

nr-reporting-summary

## Data Availability

All data generated or analyzed during this study are included in this published article and its supplementary information files. Raw sequencing data from whole exome sequencing and RNAseq have been deposited in the NCBI Sequence Read Archive (SRA) with the accession code BioProject: PRJNA1085200.

## Code availability

Analysis pipeline for genomic and transcriptomic data was carried out using the command-line tools mentioned above and was automated by means of the Nextflow v23.10 workflow system. Statistical analyses were performed in GraphPad Prism v8.0.1 and R v4.3.3. The R packages ‘ggplot2’, ‘caret’, ‘car’ and ‘pROC’ were used as previously described.
