# Supplementary material for: Increased blood draws for ultrasensitive ctDNA and CTCs detection in early breast cancer patients
Source: NPJ Breast Cancer. 2024 May 15;10:36. doi: 10.1038/s41523-024-00642-6 (PMC11096188; doi:10.1038/s41523-024-00642-6)
Supplement: Supplementary file 1 — SUPPLEMENTARY INFORMATION [file 41523_2024_642_MOESM1_ESM.pdf]

SUPPLEMENTARY INFORMATION FOR

INCREASED BLOOD DRAWS FOR ULTRASENSITIVE ctDNA AND CTCs DETECTION IN EARLY BREAST CANCER PATIENTS

Alfonso Alba-Bernal et al.

Corresponding author: Iñaki Comino-Méndez,
inaki.comino@ibima.eu

The PDF file includes:

Supplementary Figures 1 to 10

Supplementary Tables 1 to 9

SUPPLEMENTARY FIGURES (1-10)

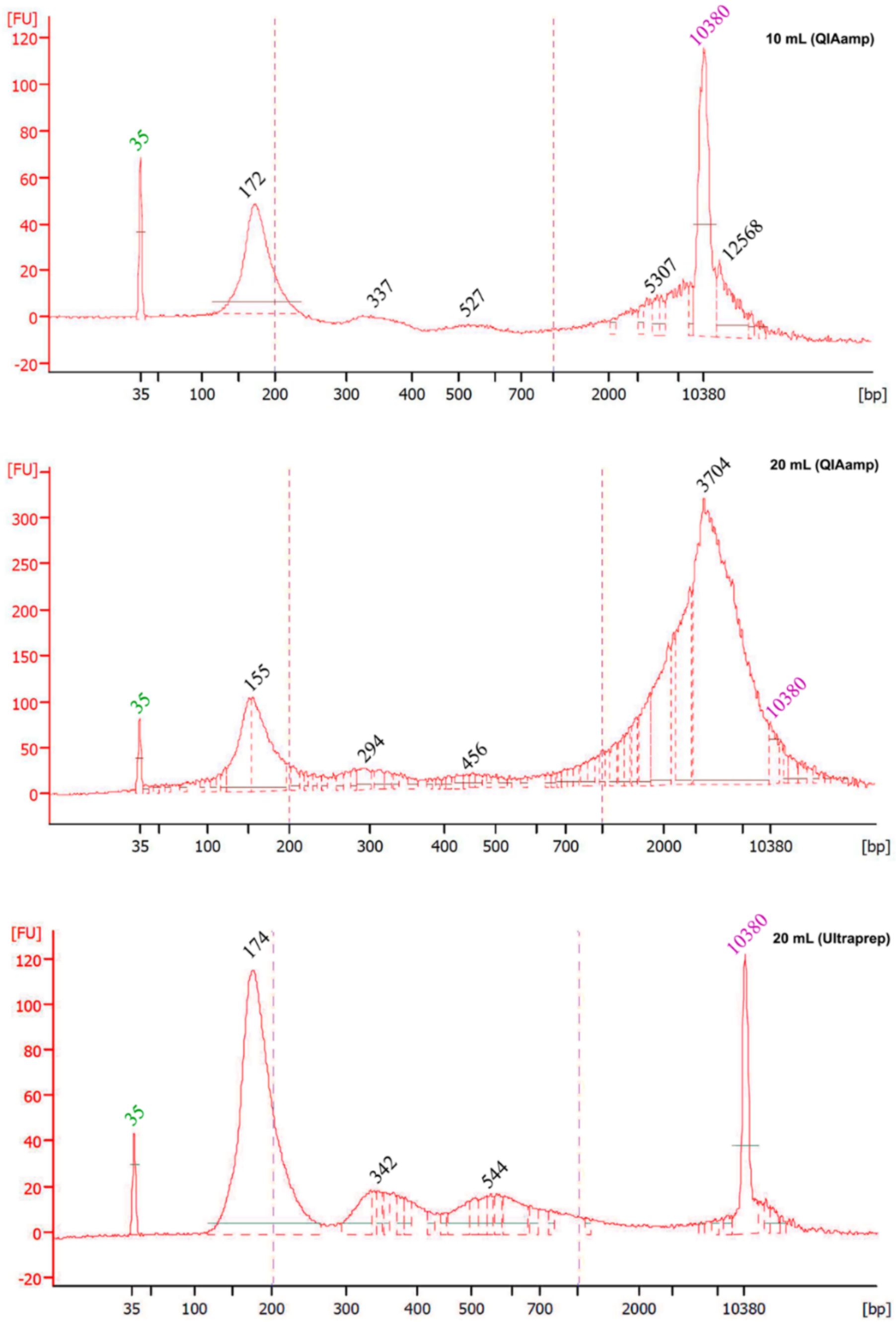

Supplementary Fig. 1. Fluorescence profiles illustrating the extraction of total circulating-free DNA (cfDNA) using varying plasma volumes. The first two graphs represent the extraction of 10 and 20 mL of plasma using the silica column-based QIAamp Circulating Nucleic Acid Kit (Qiagen). The last graph demonstrates the profile obtained through extraction using the solid version of the Ultraprep protocol.

a

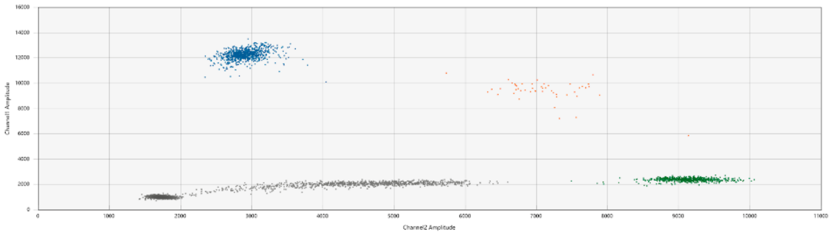

b

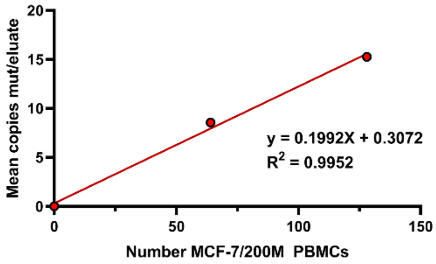

Supplementary Fig. 2. Spike-in experiment with MCF7 cells. **a)** Validation of the E545K mutation in the *PIK3CA* gene. **b)** Linear regression analysis of the spike-in experiments. Technical triplicates were conducted for each spike-in sample.

PBMCs, Peripheral blood mononuclear cells.

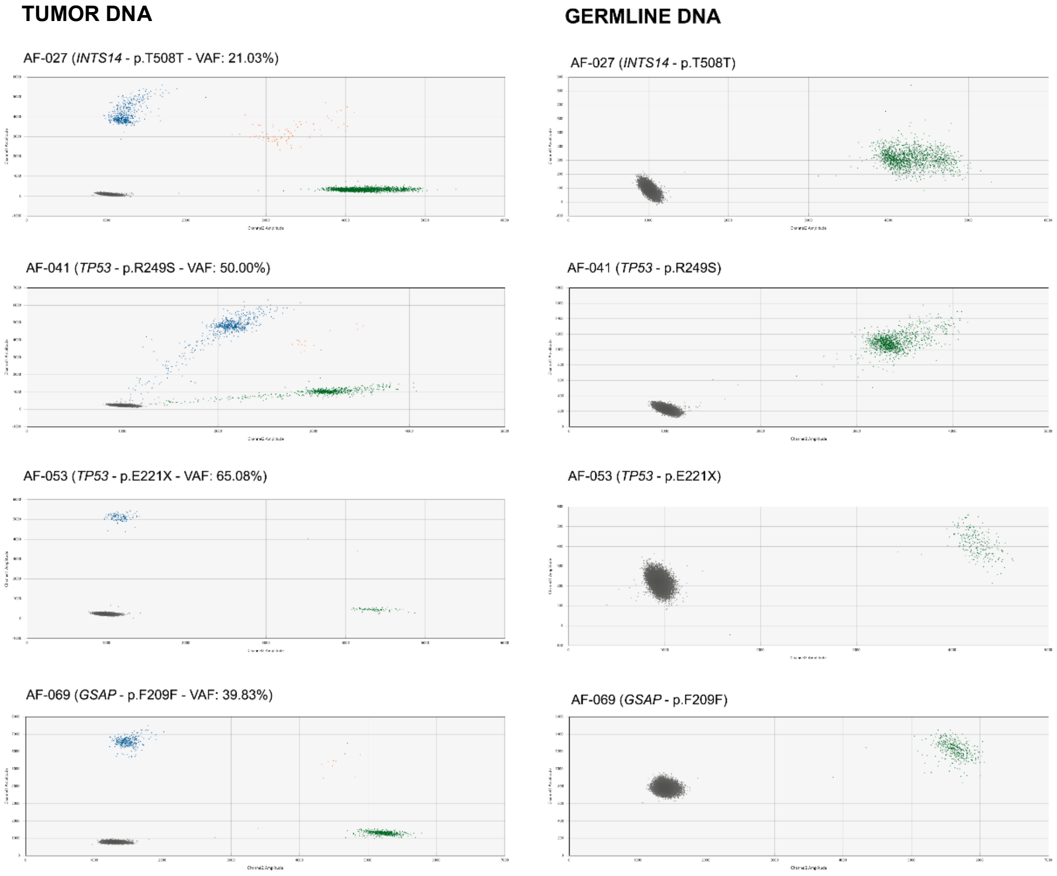

Supplementary Fig. 3. Validation of somatic mutations using ddPCR. Graphs depicting an example for the validation of four mutations identified through whole-exome sequencing (WES) in tumor and germline DNA samples. Mutant droplets are represented by blue dots (FAM-labelled), wild-type droplets by green dots (VIC-labelled), and droplets containing both wild-type and mutant molecules by orange dots.

VAF, variant allele frequency.

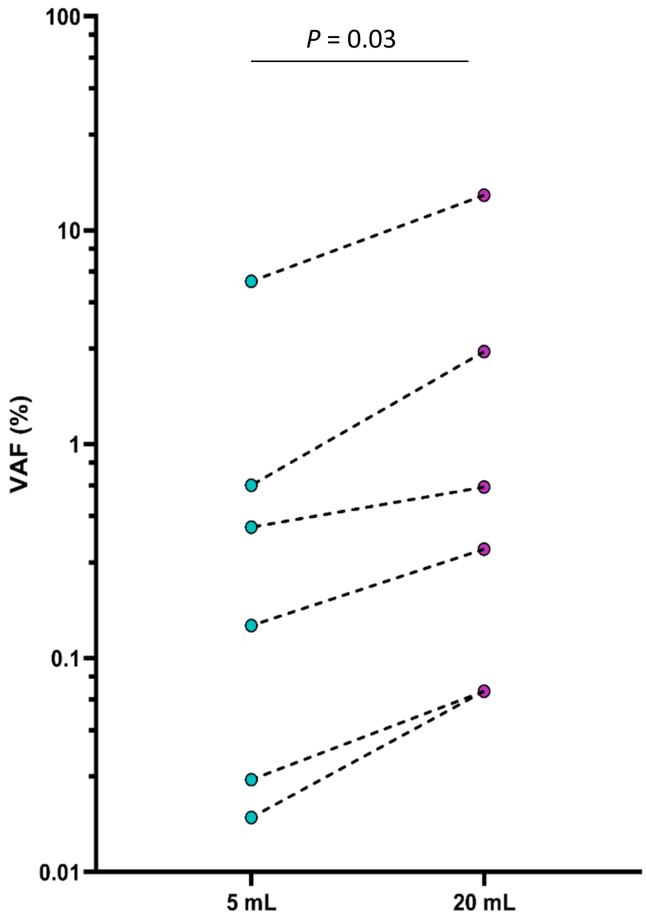

Supplementary Fig. 4. VAF of detected mutations in ctDNA using 5 and 20 mL of plasma. The graph illustrates the VAF for each mutation, and the P-value was calculated using the Wilcoxon matched-pairs test with a sample size of n=12.

VAF, variant allele frequency.

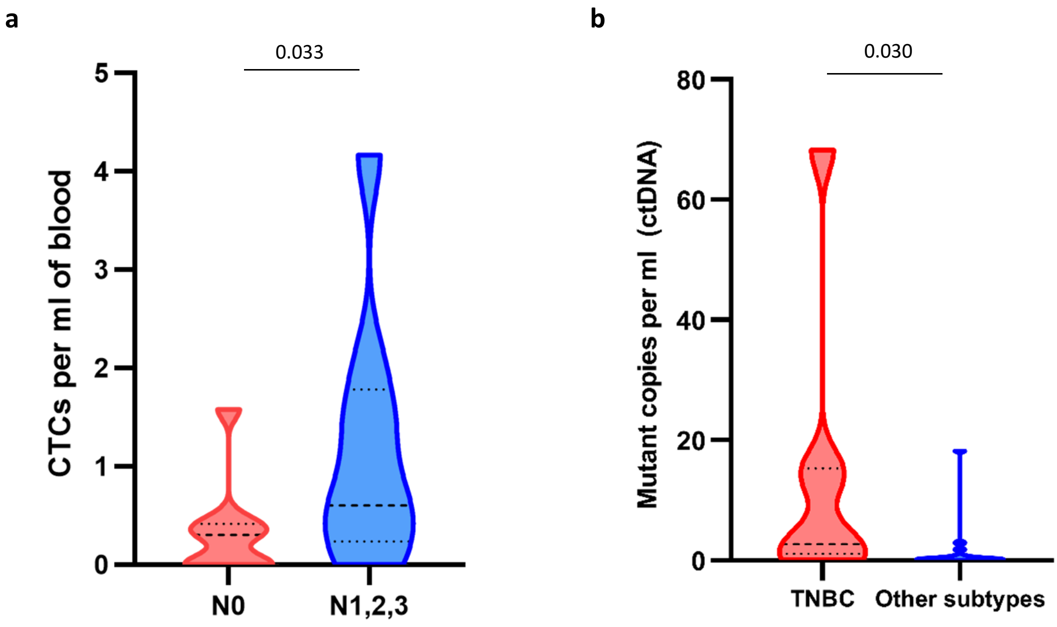

Supplementary Fig. 5. Associations between blood tumor components and clinicopathological characteristics at pre-treatment setting. a) Graph depicting the CTCs per mL of blood in patients with negative and positive-affected lymph nodes. **b)** Comparison of mutant copies per mL of plasma between TNBC cases and other subtypes. The statistical significance was determined using a Kolmogorov-Smirnov test for A (n=19) and a Mann-Whitney test for B (n=21) (Quartiles are illustrated as dots lines, median as dashed lines, and the upper and lower limits of the plots representing the maximum and minimum values, respectively).

CTCs, Circulating tumor cells; TNBC, Triple negative breast cancer; N0, no lymph nodes affected; N1-N3, from 1 to 10 lymph nodes affected.

a

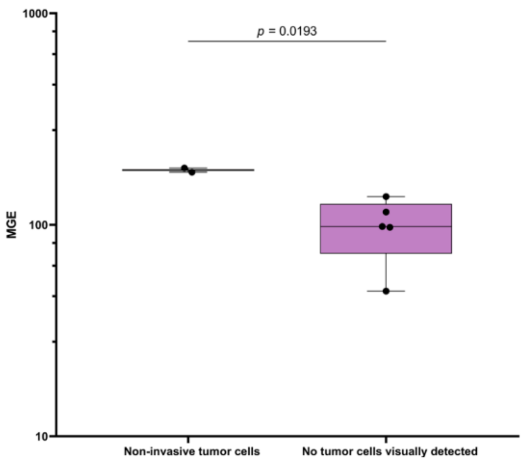

b

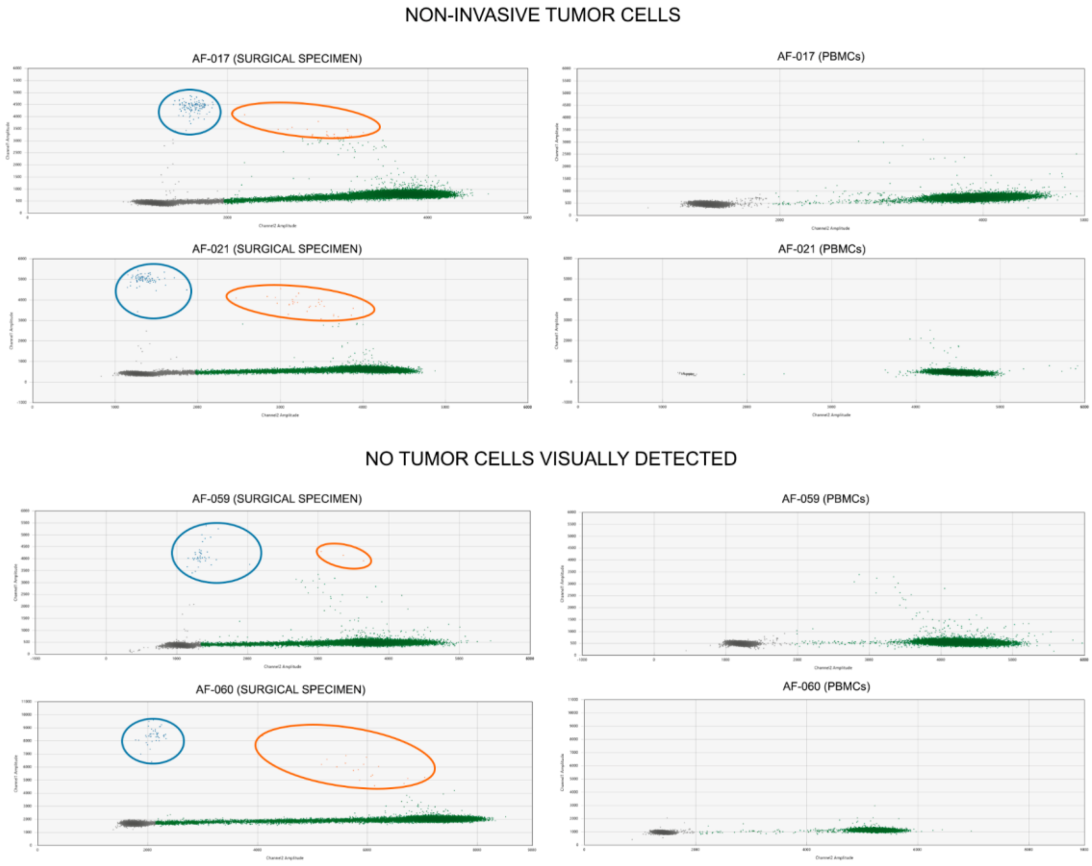

Supplementary Fig. 6. Tumor DNA detection in PCR tissues with and without in-situ tumor cells. a) Shows MGEs observed in surgical tissues with and without observable in-situ tumor cells (medians are depicted). **b)** Provides examples of ddPCR plots displaying tumor DNA detection (blue - FAM and orange - FAM/VIC droplets) in two tissues with in-situ tumor cells and two without visible tumor cells. The box-and-whiskers plots display the median, maximum, and minimum values as bars (n=2 for Non-invasive tumor cells, n=5 for No tumor cells visually detected).

MEGs, mutant genomic equivalents; ddPCR, droplet-digital PCR.

a

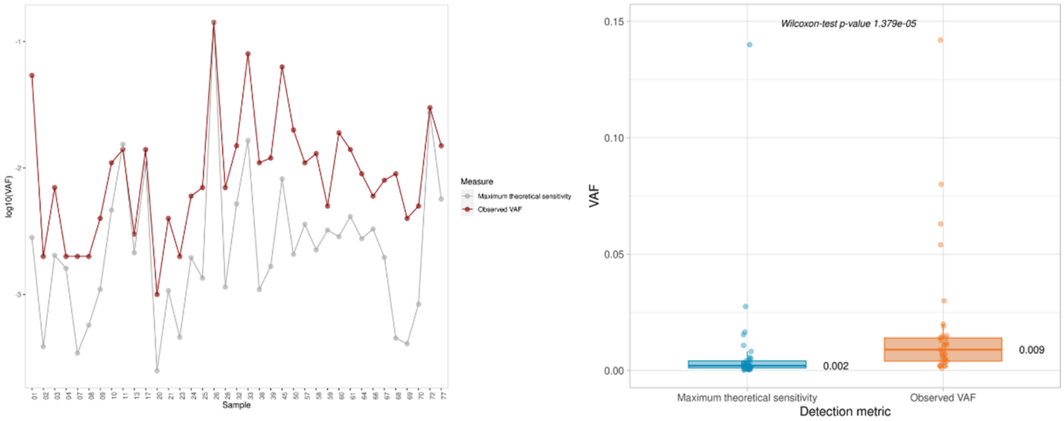

b

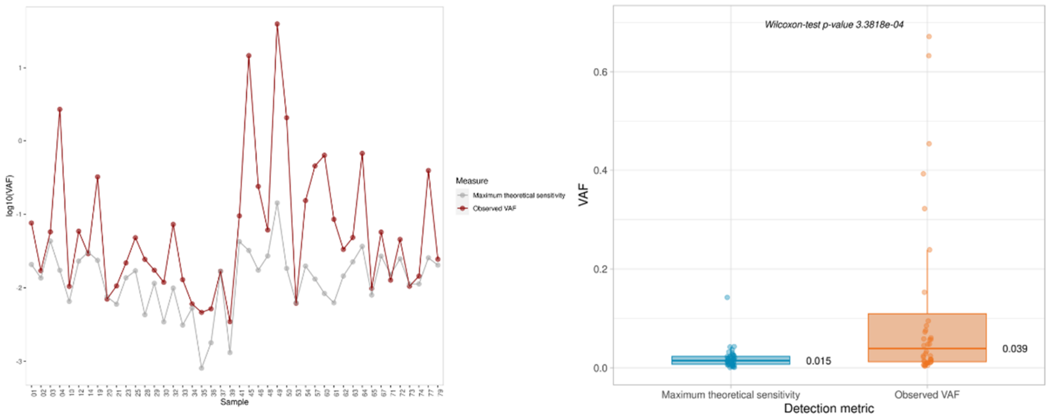

Supplementary Fig. 7. Comparison of the calculated minimum theoretical variant allele frequency (VAF) to the observed VAF for each sample in a) CTCs and **b)** ctDNA. Medians are depicted, while the boxplot whiskers define the minimum value (spanning 25% of the data from the lower whisker to the bottom of the box) and the maximum value (spanning 25% of the data from the upper whisker to the top of the box).

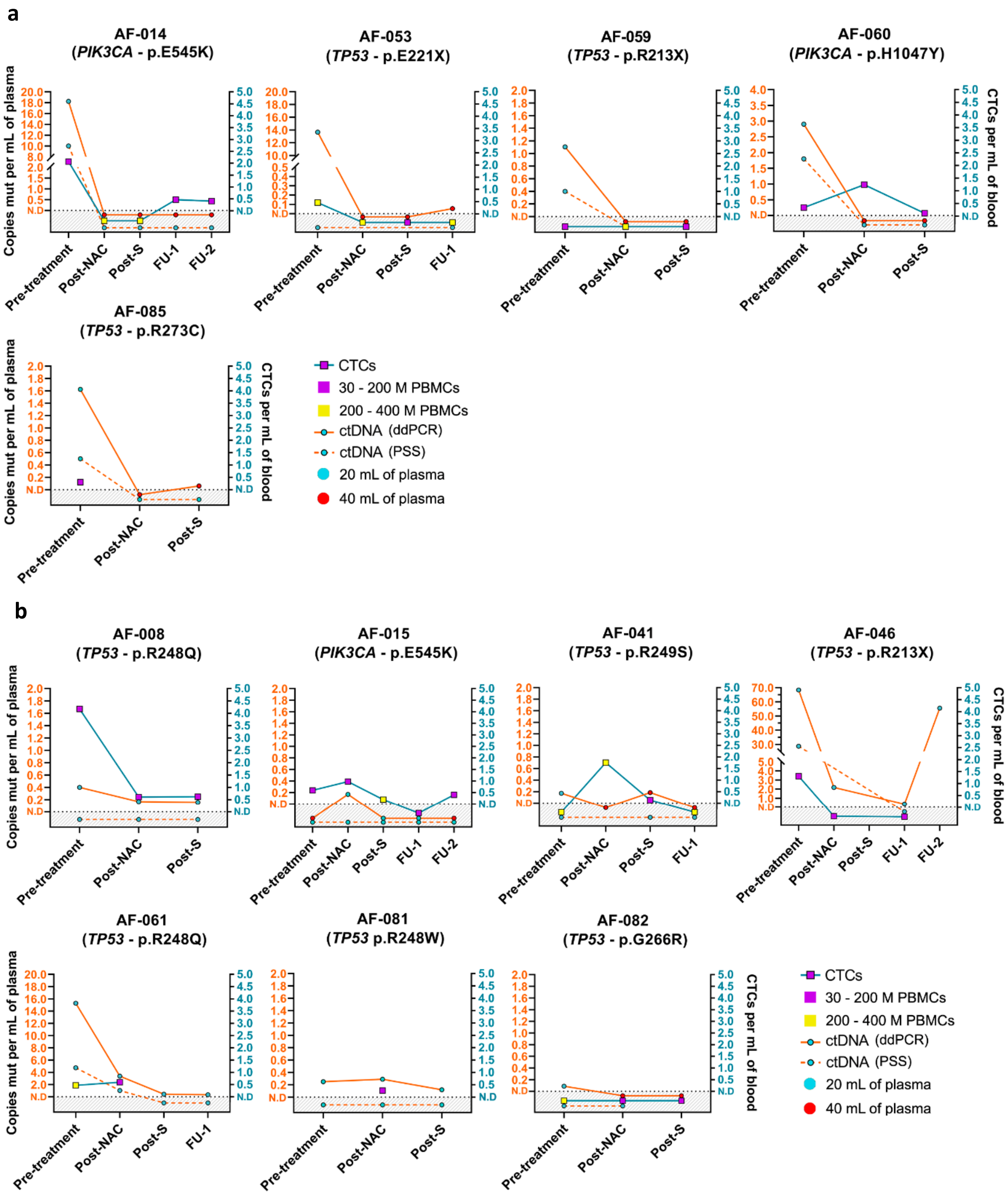

Supplementary Fig. 8. ctDNA detection comparing ddPCR and PSS in a) patients achieving PCR and **b)** without PCR.

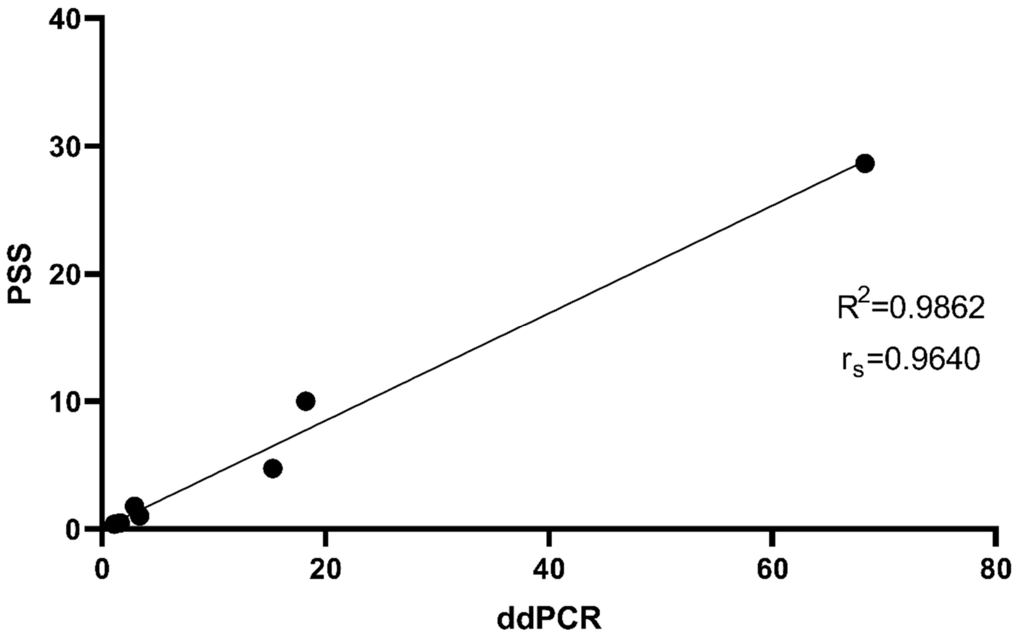

Supplementary Fig. 9. Linear regression analysis between ddPCR and PSS. The coefficient of determination (R^2) and Spearman correlation coefficient (r_s) are displayed.

PSS, Plasma-SeqSensei; ddPCR, droplet-digital PCR.

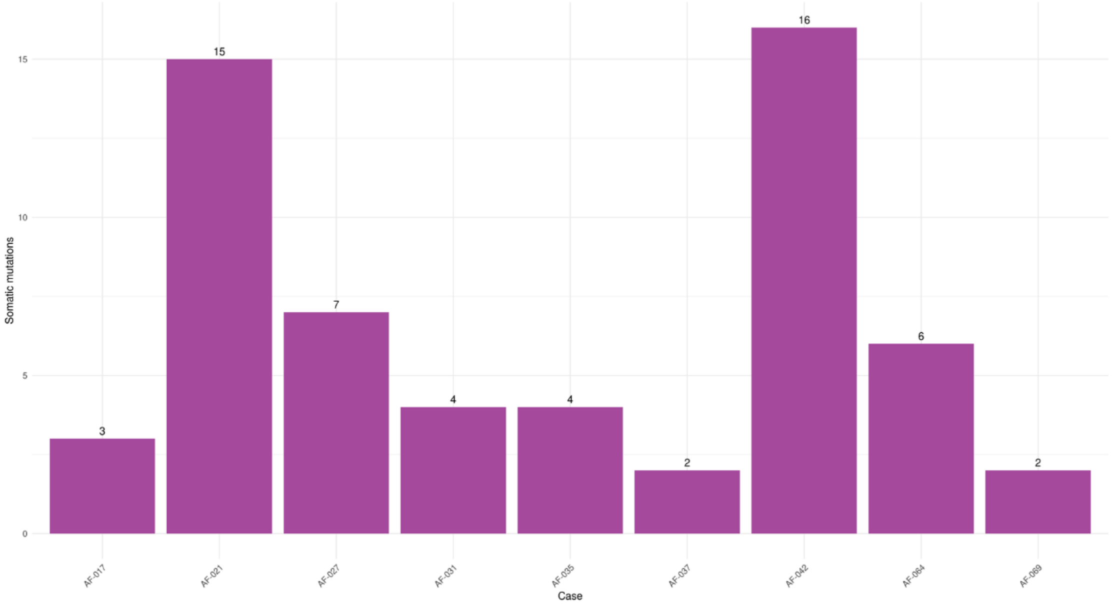

Supplementary Fig. 10. Mutations detected using a fixed NGS gene panel. The graphs depict the number of trackable mutations identified through a 33-gene panel specific to breast cancer.

SUPPLEMENTARY TABLES (1-9)

Supplementary Table 1. Details for the spike-in experiments involving MCF7 cells, including data from the three replicates utilized for logistic regression generation. *WT, Wild-type.*

	Spike-in samples	Pre-enrichment cell number (millions)	Total ng	Mutant copies/uL	WT copies/uL	Eluate volume (uL)
**FIRST REPLICATE**	0 MCF7 in 200M PBMCs	-	1956.027	0	3418	240
	64 MCF7 in 200M PBMCs	157.950	1197.227	0.057	2092	240
	128 MCF7 in 200M PBMCs	166.400	1583.909	0.065	1950	320
**SECOND REPLICATE**	0 MCF7 in 200M PBMCs	-	1208.610	0	3240	200
	64 MCF7 in 200M PBMCs	143.970	1098.763	0.047	1754	200
	128 MCF7 in 200M PBMCs	141.700	1043.757	0.062	2798	220
**THIRD REPLICATE**	0 MCF7 in 200M PBMCs	-	1619.140	0	2572	160
	64 MCF7 in 200M PBMCs	161.200	741.827	0.017	2714	160
	128 MCF7 in 200M PBMCs	166.400	1356.027	0.040	2154	280

MCF7 cell number in spike-in samples	Mean mutant copies/uL	Mean mutant copies/eluate
0	0	0
64	0.040	8.552
128	0.056	15.261

Supplementary Table 2. ctDNA and CTCs detection in the blood samples. *cfDNA, Circulating-free DNA; MGE, Mutant genomic equivalent; VAF, Variant allele frequency; PBMCS, Peripheral blood mononuclear cells; CTCs, Circulating tumor cells*

PATIENT	TIMEPOINT	PLASMA VOLUME (mL)	cfDNA ng	MGE	Mutant copies/mL plasma	Mutant copies/mL plasma	Mutant copies/mL plasma	VAF (%)	TOTAL PARTITIONS	PBMCs	CTCs ng	Calculated number of PBMCs after depletion	PMBCs depletion efficiency (log)	Mutant copies in total eluate	Mutant copies in total eluate	Calculated total CTCs	Calculated total blood	CTCs/mL blood	TOTAL PARTITIONS
					FIRST ASSAY	SECOND ASSAY								FIRST ASSAY	SECOND ASSAY				
AF-008	PRE-TREATMENT	20	31.824	733.337	0.399	-	0.399	0.076	7	133000000	233.800	35424.242	3.575	33.360	-	277.288	66.500	4.170	12
	POST-NAC	20	48.576	251.090	0.166	-	0.166	0.017	10	154700000	1704.000	258181.818	2.778	6.834	-	54.754	90.035	0.608	7
	POST-SURGERY	20	15.292	266.401	0.156	-	0.156	0.057	6	132600000	324.400	49151.515	3.431	4.020	-	31.147	50.093	0.622	22
AF-014	PRE-TREATMENT	20	38.148	31347.288	18.240	-	18.240	2.712	8	50000000	410.000	62121.212	2.906	6.132	-	48.865	23.650	2.066	14
	POST-NAC	40	54.384	0	0	0	0	0	15	256000000	1471.117	222896.515	3.060	0	0	0	153.600	0	25
	POST-SURGERY	40	75.913	0	0	0	0	0	23	219700000	147.198	22302.727	3.993	0	0	0	135.116	0	15
	FOLLOW-UP 1	40	59.700	0	0	0	0	0	19	111600000	1920.430	290974.242	2.584	10.872	-	88.630	189.720	0.467	10
	FOLLOW-UP 2	40	50.950	0	0	0	0	0	26	149400000	1153.270	174737.879	2.932	6.456	-	51.583	126.990	0.406	12
	FOLLOW-UP 3	20	10.444	791	0.054	-	0.054	0.250	6	73300000	809.551	122659.242	2.776	0	-	0	84.295	0	16
	FOLLOW-UP 4	40	109.895	0	0	0	0	0	16	60100000	374.125	56685.606	3.025	2.720	-	24.240	44.073	0.550	8
AF-015	PRE-TREATMENT	40	66.480	0	0	0	0	0	14	50375000	600.840	91036.364	2.743	3.888	-	30.039	50.375	0.596	9
	POST-NAC	20	101.112	320.397	0.169	-	0.169	0.010	10	83200000	141.840	21490.909	3.588	4.158	-	32.305	33.030	0.978	7
	POST-SURGERY	40	57.156	0	0	0	0	0	11	241200000	42.822	6488.182	4.570	0	2.504	18.429	94.872	0.194	12
	FOLLOW-UP 1	40	28.644	0	0	0	0	0	6	54275000	2500.000	378787.879	2.156	0	-	-	56.989	-	-
	FOLLOW-UP 2	40	15.959	0	0	0	0	0	13	114600000	308.820	46790.909	3.389	3.940	-	30.476	75.636	0.403	10
	FOLLOW-UP 3	40	121.153	0	0	0	0	0	16	69120000	2007.548	304173.939	2.356	0	-	0	87.552	0	16
	FOLLOW-UP 4	40	138.991	0	0	0	0	0	16	201840000	2427.479	367799.848	2.739	0	5.120	40.249	139.269	0.289	24
AF-017	PRE-TREATMENT	20	21.555	189.758	0.109	-	0.109	0.029	8	253850000	47.088	7134.515	4.551	0	0	0	126.925	0	8
	POST-NAC	40	140.102	0	0	0	0	0	16	-	-	-	-	-	-	-	-	-	-
	POST-SURGERY	40	125.796	0	0	0	0	0	16	328800000	57.662	8736.667	4.576	0	0	0	164.870	0	8
	FOLLOW-UP 1	40	34.716	0	0	0	0	0	14	130800000	61.129	9261.970	4.150	0	-	-	42.390	-	-
	FOLLOW-UP 2	40	14.583	0	0	0	0	0	6	93600000	276.354	41871.818	3.349	0	-	-	308.880	-	-
	FOLLOW-UP 3	40	17.356	0	0	0	0	0	11	-	-	-	-	-	-	-	-	-	-
	FOLLOW-UP 4	20	51.634	501	0.344	-	0.344	0.032	8	43400000	1578.544	239173.333	2.259	8.960	-	70.751	56.420	1.254	16
AF-021	PRE-TREATMENT	20	27.972	2730.358	1.778	-	1.778	0.322	14	104400000	27.794	4211.212	4.394	0	0	0	45.344	0	16
	POST-NAC	20	87.246	185.492	0.114	-	0.114	0.007	9	38400000	2653.360	402024.242	1.980	4.030	-	31.231	20.480	1.525	13
	POST-SURGERY	20	110.420	354.446	0.235	-	0.235	0.011	11	200800000	615.704	93288.485	3.333	0	2.800	20.912	90.360	0.231	32
AF-027	PRE-TREATMENT	40	35.240	0	0	0	0	0	10	-	-	-	-	-	-	-	-	-	-
	POST-NAC	40	48.312	318.677	0	0.2882	0.141	0.022	16	94900000	1437.496	217802.424	2.639	5.376	-	42.523	36.235	1.174	16
	POST-SURGERY	40	58.344	0	0	0	0	0	19	133800000	337.750	51174.242	3.417	2.666	-	19.784	50.844	0.389	16
AF-031	PRE-TREATMENT	40	38.814	563.711	0	0.3042	0.152	0.048	14	155000000	490.388	74301.197	3.319	0	4.896	38.496	63.722	0.604	27
	POST-NAC	40	39.756	0	0	0	0	0	20	66000000	4.714	714.212	4.966	6.070	-	48.345	35.829	1.349	5
	POST-SURGERY	-	-	-	-	-	-	-	-	-	-	-	-	-	-	-	-	-	-
	FOLLOW-UP 1	20	153.446	1130.185	0.278	-	0.278	0.024	6	32400000	575.520	87200.000	2.570	3.864	-	29.838	14.904	2.002	12
AF-035	PRE-TREATMENT	40	57.349	301.499	0	0.1211	0.061	0.017	19	237000000	133.723	20261.061	4.068	0	0	0	201.450	0	27
	POST-NAC	40	192.091	690.670	0	0.4272	0.214	0.012	16	229200000	221.731	33595.545	3.834	0	0	0	78.437	0	20
	POST-SURGERY	40	79.280	0	0	0	0	0	16	72600000	41.135	6232.576	4.066	0	-	-	50.820	-	-
AF-037	PRE-TREATMENT	20	66.660	1469.042	0.734	-	0.734	0.073	8	85800000	126.850	19219.697	3.650	3.936	-	30.442	20.378	1.494	8
	POST-NAC	20	212.272	828.925	0.332	-	0.332	0.013	8	104400000	39.946	6052.424	4.237	3.848	-	29.704	40.455	0.734	4
	POST-SURGERY	20	124.476	227.317	0.108	-	0.108	0.006	16	-	-	-	-	-	-	-	-	-	-
	FOLLOW-UP 1	20	818.136	1147.870	0.293	-	0.293	0.005	8	79800000	828.707	125561.667	2.803	0	-	-	99.750	-	16
	FOLLOW-UP 2	20	368.808	577.945	0.338	-	0.338	0.005	8	-	-	-	-	-	-	-	-	-	-
	FOLLOW-UP 3	20	380.812	346	0.176	-	0.176	0.003	16	-	-	-	-	-	-	-	-	-	-
AF-041	PRE-TREATMENT	20	39.732	202.200	0.172	-	0.172	0.017	8	291300000	73.167	11085.909	4.420	0	0	0	114.699	0	12
	POST-NAC	40	391.908	0	0	0	0	0	20	95900000	602.829	91337.727	3.021	0	7.664	61.717	35.163	1.755	16
	POST-SURGERY	40	502.392	526.565	0	0.3682	0.184	0.003	20	415800000	395.730	59959.091	3.841	5.232	-	41.315	308.880	0.134	9
	FOLLOW-UP 1	40	184.800	0	0	0	0	0	19	307200000	208.384	31573.333	3.988	0	0	0	157.989	0	16
AF-042	PRE-TREATMENT	20	15.576	448.246	0.230	-	0.230	0.095	7	78600000	720.720	109200.000	2.857	0	-	0	66	0	16
	POST-NAC	40	34.254	0	0	0	0	0	8	138000000	1546.990	234392.424	2.770	0	0	0	140	0	16
	POST-SURGERY	40	1778.568	0	0	0	0	0	8	46000000	468.600	71000.000	2.811	0	-	0	28	-	8
	FOLLOW-UP 1	20	4.778	0	0	-	-	0	4	58200000	324.000	49090.909	3.074	0	-	-	58.200	-	8
	FOLLOW-UP 2	20	123.153	709	0.560	-	0.560	0.019	8	-	-	-	-	-	-	-	-	-	-
AF-046	PRE-TREATMENT	20	20.550	91017.061	68.300	-	68.300	14.616	10	146250000	80.449	12189.242	4.079	12.912	-	105.744	80.730	1.310	8
	POST-NAC	20	37.998	2750.843	2.184	-	2.184	0.239	8	45600000	22.564	3418.788	4.125	0	-	-	17.936	-	-
	POST-SURGERY	-	-	-	-	-	-	-	-	-	-	-	-	-	-	-	-	-	-
	FOLLOW-UP 1	20	24.348	450.027	0.341	-	0.341	0.061	14	85200000	107.768	16328.485	3.717	0	-	-	97.980	-	-
	FOLLOW-UP 2	20	4.630	55422.194	55.500	-	55.500	39.502	5	-	-	-	-	-	-	-	-	-	-
AF-053	PRE-TREATMENT	20	35.986	22730.577	13.650	-	13.650	2.084	7	222900000	318.032	48186.667	3.665	0	9.072	73.529	159.214	0.462	16
	POST-NAC	40	77.788	0	0	0	0	0	16	218900000	253.416	38396.364	3.756	0	0	0	109.450	0	12
	POST-SURGERY	40	63.460	0	0	0	0	0	16	31500000	17.860	2706.061	4.066	0	-	-	24.413	-	-
	FOLLOW-UP 1	40	105.152	196.574	0	0.1092	0.055	0.006	14	150600000	326.804	49515.758	3.483	0	0	0	97.890	0	16
	FOLLOW-UP 2	40	39.756	0	0	0	0	0	16	46800000	1011.158	153205.758	2.485	0	-	0	42.120	0	16
	FOLLOW-UP 3	40	142.126	0	0	0	0	0	16	180240000	496.166	75176.667	3.380	0	0	0	210.280	0	16
AF-059	PRE-TREATMENT	20	33.446	1551.677	1.104	-	1.104	0.153	6	70200000	56.198	8514.848	3.916	0	-	0	35.100	0	8
	POST-NAC	40	32.925	0	0	0	0	0	11	201600000	294.930	44686.364	3.654	0	0	0	101	0	16
	POST-SURGERY	40	17.901	0	0	0	0	0	10	55800000	48.312	7320.000	3.882	0	-	-	61.380	-	-
AF-060	PRE-TREATMENT	20	50.182	6894.596	2.904	-	2.904	0.453	8	178800000	183.916	27866.061	3.807	2.688	-	19.972	59.600	0.335	8
	POST-NAC	40	114.960	0	0	0	0	0	16	103200000	292.908	44380.000	3.366	7.728	-	62.254	50.126	1.242	8
	POST-SURGERY	40	68.663	0	0	0	0	0	14	104400000	204.600	31000.000	3.527	2.512	-	18.496	167.040	0.111	8
AF-061	PRE-TREATMENT	20	78.849	15116.043	15.280	-	15.280	0.633	8	214840000	229.614	34790.000	3.791	0	5.110	40.291	85.936	0.469	18
	POST-NAC	20	105.642	2728.884	3.370	-	3.370	0.085	10	76800000	159.780	24209.091	3.501	5.616	-	44.536	74.880	0.595	8
	POST-SURGERY	20	45.667	459.711	0.432	-	0.432	0.033	9	-	-	-	-	-	-	-	-	-	-
	FOLLOW-UP 1	20	29.304	428.691	0.341	-	0.341	0.048	8	-	-	-	-	-	-	-	-	-	-
	FOLLOW-UP 3	40	248.824	0	0	0	0	0	16	10500000	452.639	68581.667	2.185	0	-	0	5.250	0	16
AF-064	PRE-TREATMENT	20	18.043	3671.875	2.672	-	2.672	0.672	8	62400000	238.673	36162.576	3.237	5.248	-	41.449	26.208	1.582	8
	POST-NAC	40	82.902	246.092	0	0.2412	0.121	0.010	14	87600000	58.257	8826.818	3.997	0	-	-	61.320	-	-
	POST-SURGERY	40	16.610	0	0	0	0	0	13	160200000	200.640	30400.000	3.722	2.416	-	17.690	256.320	0.069	8
	FOLLOW-UP 1	40	61.041	0	0	0	0	0	16	147600000	313.982	47573.030	3.492	0	-	0	265.680	0	8
AF-069	PRE-TREATMENT	20	24.554	424.935	0.227	-	0.227	0.057	8	235550000	336.311	50956.212	3.665	0	6.832	54.737	149.182	0.367	16
	POST-NAC	40	76.733	0	0	0	0	0	14	116400000	1461.124	221382.424	2.721	20.544	-	169.771	62.080	2.735	16
	POST-SURGERY	40	75.015	0	0	0	0	0	16	98400000	1617.218	245033.050	2.604	17.376	-	143.194	196.800	0.728	16
	FOLLOW-UP 1	40	123.816	0	0	0	0	0	16	115800000	788.012	119395.758	2.987	13.120	-	107.489	254.760	0.422	16
AF-081	PRE-TREATMENT	40	131.298	795.745	0.113	0.3848	0.249	0.020	16	-	-	-	-	-	-	-	-	-	-
	POST-NAC	20	26.664	367.198	0.289	-	0.289	0.045	8	57900000	23.998	3636.000	4.202	2.472	-	18.160	69.480	0.261	4
	POST-SURGERY	20	58.715	186.676	0.119	-	0.119	0.010	8	-	-	-	-	-	-	-	-	-	-
	FOLLOW-UP 1	40	781.896	0	0	0	0	0	32	-	-	-	-	-	-	-	-	-	-
AF-082	PRE-TREATMENT	40	58.504	255.741	0	0.1744	0.087	0.014	16	135000000	67.163	10176.212	4.123	0	0	0	203.780	0	12
	POST-NAC	40	114.840	0	0	0	0	0	16	88000000	475.420	72033.333	3.087	0	-	-	145.200	-	-
	POST-SURGERY	40	92.826	0	0	0	0	0	16	70200000	60.327	9140.485	3.885	0	-	-	101.790	-	-
	FOLLOW-UP 1	40	203.040	0	0	0	0	0	16	67440000	2499.529	378716.515	2.251	0	-	0	121.390	0	16
AF-085	PRE-TREATMENT	20	25.824	3072.486	1.624	-	1.624	0.393	8	114000000	116.013	17577.727	3.812	4.016	-	31.113	102.600	0.303	8
	POST-NAC	40	69.168	0	0	0	0	0	16	-	-	-	-	-	-	-	-	-	-
	POST-SURGERY	20	48.465	239.387	0	0.1192	0.060	0.016	16	-	-	-	-	-	-	-	-	-	-
	FOLLOW-UP 1	40	80.925	0	0	0	0	0	16	75840000	708.255	107311.364	2.849	0	-	0	79.632	0	16

Supplementary Table 3. Filtered WES data revealing the number of somatic mutations per patient. *WES, Whole-exome sequencing.*

Patient	Mutations PASS in WES data
**AF-015**	22
**AF-017**	10
**AF-021**	21
**AF-027**	5
**AF-031**	7
**AF-035**	14
**AF-037**	9
**AF-041**	301
**AF-042**	79
**AF-046**	15
**AF-053**	15
**AF-059**	17
**AF-060**	38
**AF-061**	12
**AF-064**	18
**AF-069**	8
**AF-081**	47
**AF-082**	17
**AF-085**	13

**Median**	15
**Range**	5-301

Supplementary Table 4. Somatic mutations selected as tumor biomarkers for ctDNA and CTCs detection. VAFs obtained in WES, RNAseq and in ddPCR re-validation are shown. *WES, Whole-exome sequencing; VAF, Variant allele frequency; ddPCR, Droplet-digital PCR.*

Patient	Gene	Mutation	WES VAF (%)	RNAseq VAF (%)	ddPCR VAF (%)
AF-008	*TP53*	p.R248Q	-	57.380	30.820
AF-014	*PIK3CA*	p.E545K	-	58.330	42.290
AF-015	*PIK3CA*	p.E545K	35.300	-	37.310
AF-017	*F13A1*	p.V297I	56.100	-	50.590
AF-021	*TSPAN32*	p.F37F	45.300	-	40.030
AF-027	*INTS14*	p.T508T	20.000	-	21.030
AF-031	*ADAM29*	p.G414E	42.400	-	38.770
AF-035	*TMEM205*	p.A183T	30.500	-	43.010
AF-037	*IPO8*	p.R53Q	31.000	-	36.910
AF-041	*TP53*	p.R249S	41.200	-	50.000
AF-042	*CTTNBP2*	p.R74L	51.000	-	40.440
AF-046	*TP53*	p.R213X	60.100	-	57.510
AF-053	*TP53*	p.E221X	51.300	-	65.080
AF-059	*TP53*	p.R213X	83.000	-	83.480
AF-060	*PIK3CA*	p.H1047Y	11.700	-	12.700
AF-061	*TP53*	p.R248Q	24.100	-	36.000
AF-064	*HIPR1*	p.R252Q	45.900	-	42.860
AF-069	*GSAP*	p.F209F	37.100	-	39.830
AF-081	*TP53*	p.R248W	40.100	-	38.750
AF-082	*TP53*	p.G266R	32.900	-	35.590
AF-085	*TP53*	p.R273C	75.500	-	71.720

Supplementary Table 5. Mutation detection using conventional and increased plasma volumes in a subset of pre-treatment samples. *ddPCR, Droplet-digital PCR; ctDNA, Circulating tumor DNA; VAF, Variant Allele Frequency.*

Plasma sample (T1)	Mutation	5 mL of plasma	ctDNA VAF (%)	20 mL of plasma	20 mL of plasma	ctDNA VAF (%)
		ddPCR		ddPCR		
**AF-008**	*TP53* (p.R248Q)	POSITIVE	0.018	POSITIVE	-	0.070
**AF-014**	*PIK3CA* (p.E545K)	POSITIVE	0.643	POSITIVE	-	2.710
**AF-017**	*FI3AI* (p.V297I)	NEGATIVE	-	POSITIVE	-	0.020
**AF-021**	*TSPAN32* (p.F37F)	POSITIVE	0.142	POSITIVE	-	0.323
**AF-037**	*IPO8* (p.R53Q)	POSITIVE	0.027	POSITIVE	-	0.070
**AF-031**	*ADAM29* (p.G414E)	NEGATIVE	-	NEGATIVE	POSITIVE	0.040
**AF-041**	*TP53* (p.R249S)	NEGATIVE	-	POSITIVE	-	0.010
**AF-046**	*TP53* (p.R213X)	POSITIVE	5.772	POSITIVE	-	14.610
**AF-061**	*TP53* (p.R248Q)	POSITIVE	0.409	POSITIVE	-	0.630

Supplementary Table 6. ctDNA detection comparing ddPCR and PSS. *ctDNA, Circulating tumor DNA; ddPCR, Droplet-digital PCR; PSS, Plasma-SeqSensei; VAF, Variant allele frequency; CHIP, Clonal hematopoiesis of indeterminate potential; WES, Whole-exome sequencing.*

	Patient	Timepoint	ctDNA detection with selected ddPCR mutation					Additional mutation in PSS	VAF (%)	CHIP mutation	Detected in tumor sequencing (WES/RNAseq)
			Deteccion by ddPCR	copies mut/mL plasma	Detection by PSS	cfDNA ng	copies mut/mL plasma				
**PCR**	**AF-014**	PRE-TREATMENT	Yes	18.240	Yes	40.656	10.000	TP53 p.S241A	3.07	No	Yes
		POST-NAC	No	0	No	12.6192	0	-	-	-	-
		POST-SURGERY	No	0	No	45.336	0	-	-	-	-
		FOLLOW-UP 1	No	0	No	36.696	0	-	-	-	-
		FOLLOW-UP 2	No	0	No	14.084	0	-	-	-	-
	**AF-053**	PRE-TREATMENT	Yes	13.650	No	27.060	0	-	-	-	-
		FOLLOW-UP 1	Yes	0.109	No	45.632	0	-	-	-	-
	**AF-059**	PRE-TREATMENT	Yes	1.104	Yes	41.316	0.400	*TP53* p.P72H	0.1	No	No
		POST-NAC	No	0	No	65.540	0	-	-	-	-
	**AF-060**	PRE-TREATMENT	Yes	2.904	Yes	38.808	1.800	*TP53* p.E339*	0.39	No	Yes
		POST-NAC	No	0	No	80.000	0	-	-	-	-
		POST-SURGERY	No	0	No	31.421	0	-	-	-	-
	**AF-085**	PRE-TREATMENT	Yes	1.624	Yes	30.888	0.500	-	-	-	-
		POST-NAC	No	0	No	51.470	0	-	-	-	-
		POST-SURGERY	Yes	0.016	No	0	0	-	-	-	-
**NO PCR**	**AF-008**	PRE-TREATMENT	Yes	0.399	No	22.572	0	-	-	-	-
		POST-NAC	Yes	0.166	No	44.088	0	-	-	-	-
		POST-SURGERY	Yes	0.156	No	10.5336	0	-	-	-	-
	**AF-015**	PRE-TREATMENT	No	0	No	51.612	0	-	-	-	-
		POST-NAC	Yes	0.169	No	32.736	0	-		-	-
		POST-SURGERY	No	0	No	24.552	0	-		-	-
		FOLLOW-UP 1	No	0	No	31.416	0	-		-	-
		FOLLOW-UP 2	No	0	No	18.414	0	-		-	-
	**AF-041**	PRE-TREATMENT	Yes	0.172	No	53.592	0	*TP53* p.Q136P	0.12	Yes	No
								TP53 c.376-2A>G	0.17	Yes	No
		POST-SURGERY	Yes	0.3682	No	386.550	0	-		-	-
		FOLLOW-UP 1	No	0	No	103.751	0	-		-	-
	**AF-046**	PRE-TREATMENT	Yes	68.300	Yes	16.896	28.650	-	-	-	-
		FOLLOW-UP 1	Yes	0.341	No	23.998	0	-		-	-
	**AF-061**	PRE-TREATMENT	Yes	15.280	Yes	55.968	4.750	-	-	-	-
		POST-NAC	Yes	3.370	Yes	80.000	1.030	-	-	-	-
		POST-SURGERY	Yes	0.432	No	27.524	0	-		-	-
		FOLLOW-UP 1	Yes	0.341	No	32.860	0	-		-	-
	**AF-081**	PRE-TREATMENT	Yes	0.113	No	62.790	0	-	-	-	-
		POST-NAC	Yes	0.289	No	55.810	0	-	-	-	-
		POST-SURGERY	Yes	0.020	No	112.361	0	-		-	-
	**AF-082**	PRE-TREATMENT	Yes	0.087	No	67.980	0	-	-	-	-
		POST-NAC	No	0	No	74.770	0	-	-	-	-

Supplementary Table 7. Annealing temperature of ddPCR assays.

	Gene	Mutation	Annealing temperature
**AF-008**	*TP53*	p.R248Q	60°C
**AF-014**	*PIK3CA*	p.E545K	64°C
**AF-015**	*PIK3CA*	p.E545K	64°C
**AF-017**	*F13A1*	p.V297I	60°C
**AF-021**	*TSPAN32*	p.F37F	64°C
**AF-027**	*INTS14*	p.T508T	60°C
**AF-031**	*ADAM29*	p.G414E	64°C
**AF-035**	*TMEM205*	p.A183T	64°C
**AF-037**	*IPO8*	p.R53Q	60°C
**AF-041**	*TP53*	p.R249S	64°C
**AF-042**	*CTTNBP2*	p.R74L	60°C
**AF-046**	*TP53*	p.R213X	60°C
**AF-053**	*TP53*	p.E221X	60°C
**AF-059**	*TP53*	p.R213X	60°C
**AF-060**	*PIK3CA*	p.H1047Y	60°C
**AF-061**	*TP53*	p.R248Q	60°C
**AF-064**	*HIPR1*	p.R252Q	60°C
**AF-069**	*GSAP*	p.F209F	60°C
**AF-081**	*TP53*	p.R248W	60°C
**AF-082**	*TP53*	p.G266R	60°C
**AF-085**	*TP53*	p.R273C	60°C

Supplementary Table 8. Negative control tests for ddPCR assays for ctDNA and CTCs detection. Number of tests performed with germline DNA from each patient per assay to assess specificity.

Patients	Gene	Protein change	Total number of specificity tests conducted initially	Number of extra specificity tests in samples with ≤3 FAM droplets	Total number of specificity tests	Number of specificity tests without **ANY** positive doplets	Number of specificity tests with **one** positive doplet	Number of specificity tests with **two** positive doplets
**AF-008, AF-061**	*TP53*	p.R248Q	16	6	22	19	3	0
**AF-014, AF-015**	*PIK3CA*	p.E545K	43	14	57	50	7	0
**AF-017**	*F13A1*	p.V297I	19	4	23	19	3	**1**
**AF-021**	*TSPAN32*	p.F37F	8	6	14	11	3	0
**AF-027**	*INTS14*	p.T508T	8	0	8	6	2	0
**AF-031**	*ADAM29*	p.G414E	9	4	13	11	2	0
**AF-035**	*TMEM205*	p.A183T	11	2	13	12	1	0
**AF-037**	*IPO8*	p.R53Q	9	8	17	13	3	**1**
**AF-041**	*TP53*	p.R249S	14	10	24	24	0	0
**AF-042**	*CTTNBP2*	p.R74L	12	8	20	20	0	0
**AF-046, AF-059**	*TP53*	p.R213X	16	2	18	16	1	**1**
**AF-053**	*TP53*	p.E221X	20	2	22	20	2	0
**AF-060**	*PIK3CA*	p.H1047Y	8	4	12	10	2	0
**AF-064**	*HIPR1*	p.R252Q	11	4	15	15	0	0
**AF-069**	*GSAP*	p.F209F	12	2	14	12	2	0
**AF-081**	*TP53*	p.R248W	7	8	15	12	2	**1**
**AF-082**	*TP53*	p.G266R	13	2	15	11	4	0
**AF-085**	*TP53*	p.R273C	9	6	15	14	0	**1**
	**Total N (%)**		**245**	**92**	**337**	**87.53709199**	**10.97922849**	**1.483679525**

NEGATIVE CONTROLS SHOWING NEGATIVITY >98%

Supplementary Table 9. Targets regions for PSS BC RUO Kit

Gene ID	Transcript ID	CDS start	CDS end
AKT1	ENST00000554581	47	69
ERBB2	ENST00000269571	907	947
ERBB2	ENST00000269571	2,308	2,360
ERBB2	ENST00000269571	2,258	2,307
ESR1	ENST00000440973	1,108	1,143
ESR1	ENST00000440973	1,378	1,420
ESR1	ENST00000440973	1,583	1,614
KRAS	ENST00000256078	8	43
PIK3CA	ENST00000263967	254	278
PIK3CA	ENST00000263967	329	352
PIK3CA	ENST00000263967	353	367
PIK3CA	ENST00000263967	1,033	1,058
PIK3CA	ENST00000263967	1,085	1,115
PIK3CA	ENST00000263967	1,252	1,264
PIK3CA	ENST00000263967	1,348	1,387
PIK3CA	ENST00000263967	1611	1659
PIK3CA	ENST00000263967	2,138	2,184
PIK3CA	ENST00000263967	3118	3169
TP53	ENST00000269305	144	232
TP53	ENST00000269305	293	375
TP53	ENST00000269305	376	423
TP53	ENST00000269305	451	537
TP53	ENST00000269305	574	659
TP53	ENST00000269305	695	782
TP53	ENST00000269305	783	856
TP53	ENST00000269305	888	919
TP53	ENST00000269305	920	993
TP53	ENST00000269305	994	1,080
