# Supplementary material for: Increased blood draws for ultrasensitive ctDNA and CTCs detection in early breast cancer patients
Source: NPJ Breast Cancer. 2024 May 15;10:36. doi: 10.1038/s41523-024-00642-6 (PMC11096188; doi:10.1038/s41523-024-00642-6)
Supplement: Supplementary file 2 — nr-reporting-summary [file 41523_2024_642_MOESM2_ESM.pdf]

Corresponding author(s): Iñaki Comino-Méndez

Last updated by author(s): Mar 7, 2024

Reporting Summary

Nature Portfolio wishes to improve the reproducibility of the work that we publish. This form provides structure for consistency and transparency in reporting. For further information on Nature Portfolio policies, see our Editorial Policies and the Editorial Policy Checklist.

Statistics

For all statistical analyses, confirm that the following items are present in the figure legend, table legend, main text, or Methods section.

n/a	Confirmed	
☐	☒	The exact sample size (*n*) for each experimental group/condition, given as a discrete number and unit of measurement
☐	☒	A statement on whether measurements were taken from distinct samples or whether the same sample was measured repeatedly
☐	☒	The statistical test(s) used AND whether they are one- or two-sided<br>*Only common tests should be described solely by name; describe more complex techniques in the Methods section.*
☒	☐	A description of all covariates tested
☐	☒	A description of any assumptions or corrections, such as tests of normality and adjustment for multiple comparisons
☐	☒	A full description of the statistical parameters including central tendency (e.g. means) or other basic estimates (e.g. regression coefficient) AND variation (e.g. standard deviation) or associated estimates of uncertainty (e.g. confidence intervals)
☐	☒	For null hypothesis testing, the test statistic (e.g. *F*, *t*, *r*) with confidence intervals, effect sizes, degrees of freedom and *P* value noted<br>*Give P values as exact values whenever suitable.*
☒	☐	For Bayesian analysis, information on the choice of priors and Markov chain Monte Carlo settings
☒	☐	For hierarchical and complex designs, identification of the appropriate level for tests and full reporting of outcomes
☒	☐	Estimates of effect sizes (e.g. Cohen's *d*, Pearson's *r*), indicating how they were calculated

Our web collection on statistics for biologists contains articles on many of the points above.

Software and code

Policy information about availability of computer code

Data collection	No software was used
Data analysis	No software was used

For manuscripts utilizing custom algorithms or software that are central to the research but not yet described in published literature, software must be made available to editors and reviewers. We strongly encourage code deposition in a community repository (e.g. GitHub). See the Nature Portfolio guidelines for submitting code & software for further information.

Data

Policy information about availability of data

All manuscripts must include a data availability statement. This statement should provide the following information, where applicable:

- Accession codes, unique identifiers, or web links for publicly available datasets
- A description of any restrictions on data availability
- For clinical datasets or third party data, please ensure that the statement adheres to our policy

All data generated or analyzed during this study are included in this published article and its supplementary information files. Raw sequencing data from whole exome sequencing and RNAseq have been deposited in the NCBI Sequence Read Archive (SRA) with the accession code BioProject:PRJNA1085200.

Research involving human participants, their data, or biological material

Policy information about studies with human participants or human data. See also policy information about sex, gender (identity/presentation), and sexual orientation and race, ethnicity and racism.

Reporting on sex and gender	In this scientific study focusing on breast cancer, reporting on sex and gender is not required as the research is specifically focused on a condition that primarily affects individuals assigned female at birth.
Reporting on race, ethnicity, or other socially relevant groupings	In this breast cancer study, it is pertinent to note that no reporting on race, ethnicity, or other socially relevant groupings has been conducted. The focus of the study is primarily on the specific aspects related to breast cancer, and no analysis or consideration has been made with regard to these demographic factors.
Population characteristics	The population characteristics of this study encompass women diagnosed with early breast cancer. The manuscript provides comprehensive information on various features, including age and treatment regimens, to enhance a thorough understanding of the study cohort.
Recruitment	Patients were recruited strictly based on the selection criteria outlined in the manuscript, and no biases have been considered as influencing factors in this study. The recruitment process adheres to a rigorous framework to ensure the integrity and objectivity of the research findings.
Ethics oversight	"Servicio Andaluz de Salud (SAS) – Consejería de Salud"

Note that full information on the approval of the study protocol must also be provided in the manuscript.

Field-specific reporting

Please select the one below that is the best fit for your research. If you are not sure, read the appropriate sections before making your selection.

☒ Life sciences ☐ Behavioural & social sciences ☐ Ecological, evolutionary & environmental sciences

For a reference copy of the document with all sections, see nature.com/documents/nr-reporting-summary-flat.pdf

Life sciences study design

All studies must disclose on these points even when the disclosure is negative.

Sample size	In this study, no sample-size calculation was undertaken. As a proof-of-principle study and considering the unique characteristics of the extracted samples, coupled with insights from prior research in the field, we assert that the number of patients included in the study is deemed sufficient to demonstrate the efficacy of the reported methodology in reaching our conclusions.
Data exclusions	Data exclusions for each analysis are thoroughly detailed throughout the manuscript and visually represented in the main Figure 1E, explicitly outlining the reasons for exclusion in the corresponding statistical tests.
Replication	In this study, the determinations for ctDNA and CTCs detection in a given sample could not be replicated due to the unique nature of the blood samples. Conversely, for the generation of the CTCs model using MCF7 cells, experiments were conducted in triplicates, as explicitly outlined in the manuscript.
Randomization	Randomization was deemed not relevant to our study, as we intentionally recruited patients with all breast cancer subtypes. The objective was to assess cancer detection without introducing bias, and therefore, a randomized approach was not considered necessary for the scope and design of our investigation.
Blinding	In this study, the detection of ctDNA and CTCs in blood was carried out blindly, in accordance with the prospective nature of the investigation. Clinical status assessments were conducted every six months for all patients, without consideration of the molecular determinations made in our study.

Reporting for specific materials, systems and methods

We require information from authors about some types of materials, experimental systems and methods used in many studies. Here, indicate whether each material, system or method listed is relevant to your study. If you are not sure if a list item applies to your research, read the appropriate section before selecting a response.

Materials & experimental systems

n/a	Involved in the study
☒	☐ Antibodies
☒	☐ Eukaryotic cell lines
☒	☐ Palaeontology and archaeology
☒	☐ Animals and other organisms
☒	☐ Clinical data
☒	☐ Dual use research of concern
☒	☐ Plants

Methods

n/a	Involved in the study
☒	☐ ChIP-seq
☒	☐ Flow cytometry
☒	☐ MRI-based neuroimaging

Plants

Seed stocks

Report on the source of all seed stocks or other plant material used. If applicable, state the seed stock centre and catalogue number. If plant specimens were collected from the field, describe the collection location, date and sampling procedures.

Novel plant genotypes

Describe the methods by which all novel plant genotypes were produced. This includes those generated by transgenic approaches, gene editing, chemical/radiation-based mutagenesis and hybridization. For transgenic lines, describe the transformation method, the number of independent lines analyzed and the generation upon which experiments were performed. For gene-edited lines, describe the editor used, the endogenous sequence targeted for editing, the targeting guide RNA sequence (if applicable) and how the editor was applied.

Authentication

Describe any authentication procedures for each seed stock used or novel genotype generated. Describe any experiments used to assess the effect of a mutation and, where applicable, how potential secondary effects (e.g. second site T-DNA insertions, mosiacism, off-target gene editing) were examined.
